# The Hausdorff and dynamical dimensions of self-affine sponges: a dimension gap result

**DOI:** 10.1007/s00222-017-0725-5

**Published:** 2017-04-26

**Authors:** Tushar Das, David Simmons

**Affiliations:** 1 Department of Mathematics and Statistics, University of Wisconsin – La Crosse, 1725 State Street, La Crosse, WI 54601 USA; 2 Department of Mathematics, University of York, Heslington, York, YO10 5DD UK

**Keywords:** Primary 37C45, 37C40, Secondary 37D35, 37D20

## Abstract

We construct a self-affine sponge in $\mathbb {R}^3$ whose dynamical dimension, i.e. the supremum of the Hausdorff dimensions of its invariant measures, is strictly less than its Hausdorff dimension. This resolves a long-standing open problem in the dimension theory of dynamical systems, namely whether every expanding repeller has an ergodic invariant measure of full Hausdorff dimension. More generally we compute the Hausdorff and dynamical dimensions of a large class of self-affine sponges, a problem that previous techniques could only solve in two dimensions. The Hausdorff and dynamical dimensions depend continuously on the iterated function system defining the sponge, implying that sponges with a dimension gap represent a nonempty open subset of the parameter space.

## Introduction

A fundamental question in dynamics is to find “natural” invariant measures on the phase space of a dynamical system. Such measures afford a window into the dynamical complexity of chaotic systems by allowing one to study the statistical properties of the system via observations of “typical” orbits. For example, the knowledge that Gauss measure on [0, 1] is ergodic and invariant with respect to the Gauss map allows one to compute the distribution of continued fraction partial quotients of Lebesgue almost every real number [15, §3.2]. In general, ergodic invariant measures that are absolutely continuous to Lebesgue measure are often considered the most physically relevant, since they describe the statistical properties of the forward orbits of a set of points of positive Lebesgue measure.

However, in many cases there are no invariant measures absolutely continuous to Lebesgue measure. In this circumstance, there are other ways of deciding which invariant measure is the most “natural”—for example, Sinai, Ruelle, and Bowen considered a class of invariant measures (now known as SRB measures) that still describe the behavior of forward orbits of points typical with respect to Lebesgue measure, even though these invariant measures are not necessarily absolutely continuous to Lebesgue measure, see e.g. [56]. However, there are some disadvantages to this class of measures, for example we may want to consider measures supported on a fractal subset of interest such as a basic set or a repeller, and SRB measures may not be supported on such a fractal.

A complementary approach is to judge how natural a measure is in terms of its Hausdorff dimension. For example, Lebesgue measure has the largest possible Hausdorff dimension of any measure, equal to the Hausdorff dimension of the entire space. If we are looking for measures supported on a fractal subset, it makes sense to look for one whose Hausdorff dimension is equal to the Hausdorff dimension of that set. An ergodic invariant measure with this property can be thought of as capturing the “typical” dynamics of points on the fractal. In cases where such a measure is known to exist, it is often unique; see e.g. [43, Theorem 9.3.1] and [32, Theorem 4.4.7], where this is proven in the cases of conformal expanding repellers and conformal graph directed Markov systems, respectively.

On the other hand, if the Hausdorff dimension of an invariant measure is strictly less than the Hausdorff dimension of the entire fractal, then the set of typical points for the measure is much smaller than the set of atypical points, and therefore the dynamics of “most” points on the fractal are not captured by the measure. Even so, we can ask whether the Hausdorff dimension of the fractal can be approximated by the Hausdorff dimensions of invariant measures, i.e. whether it is equal to the supremum of the Hausdorff dimensions of such measures. We call the latter number the *dynamical dimension* of the system; cf. [14], [43, §§12.2–12.3], though we note that the definition of the dynamical dimension in these references is slightly different from ours.

The question of which dynamical systems have ergodic invariant measures of full Hausdorff dimension has generated substantial interest over the past few decades, see e.g. [4, 9, 17, 18, 21, 24, 25, 27, 30–33, 42, 45, 52, 55], as well as the survey articles [7, 13, 22, 51] and the books [5, 6]. Most of the results are positive, proving the existence and uniqueness of a measure of full dimension under appropriate hypotheses on the dynamical system.

The theory in the case of (compact) expanding systems that are conformal or essentially one-dimensional is, in a sense, the most complete—the Hausdorff and box dimensions of the repeller coincide, and there exists a unique ergodic invariant full dimension measure. The equality of dimension characteristics as well as the existence of a full dimension measure is a consequence of *Bowen’s formula* in the thermodynamic formalism, which equates the Hausdorff dimension of the repeller with the unique zero of a pressure functional, see e.g. [43, Corollary 9.1.7], [23], or [49, Theorem 2.1] for an elementary proof. The uniqueness of the full dimension measure follows from the *Volume Lemma*, which describes how to compute the Hausdorff dimension of an arbitrary ergodic invariant measure, see e.g. [43, Theorems 9.1.11 and 9.3.1]. On the other hand, if either of the assumptions of compactness and expansion is dropped, then a full dimension measure may not exist, see [2, 53] respectively.

Another class of examples for which a great deal of theory has been established is the case of two-dimensional Axiom A diffeomorphisms. Loosely speaking, Axiom A diffeomorphisms are those in which there is a dichotomy between “expanding” directions and “contracting” directions, see e.g. [12] for a beautiful introduction. McCluskey and Manning [33] showed that “most” two-dimensional Axiom A diffeomorphisms have basic sets whose Hausdorff dimension is strictly greater than their dynamical dimension (i.e. the supremal dimension of invariant measures), and in particular there are no invariant measures of full dimension. So in the (topologically) generic case there can be no theory of full dimension measures. There is also a simple sufficient condition (not satisfied generically) for the existence of full dimension measures for two-dimensional Axiom A diffeomorphisms, see [21, Theorem 1.10]. This condition is also necessary, at least in the case where the system is topologically conjugate to a topologically mixing shift space, as can be seen by combining [8, p.99] with [12, Theorem 1.28].

Progress beyond these cases, and in particular in the case where the system is expanding but may have different rates of expansion in different directions, has been much slower and of more limited scope, see e.g. [7, 13, 22, 51]. Such systems, called “expanding repellers”, form another large and much-studied class of examples. They can be formally defined as follows:

### Definition 1.1

An *expanding repeller* is a dynamical system $f:K \rightarrow K$, where *K* is a compact subset of a Riemannian manifold *M*, $U \subset M$ is a neighborhood of *K*, and $f:U\rightarrow M$ is a $C^1$ transformation such that

- $$f^{-1}(K) = K$$; and
- for some *n*, $f^n$ is infinitesimally expanding on *K* with respect to the Riemannian metric.

The following question regarding such systems, stated by Schmeling and Weiss to be “one of the major open problems in the dimension theory of dynamical systems” [51, p.440], dates back to at least the early 1990s and can be found reiterated in several places in the literature by various experts in the field (see Lalley and Gatzouras [28, p.4], Kenyon and Peres [26, Open Problem], Gatzouras and Peres [22, Problem 1], Gatzouras and Peres [23, Conjecture on p.166], Peres and Solomyak [39, Question 5.1], Schmeling and Weiss [51, p.440], Petersen [40, p.188], Chen and Pesin [13, p.R108], Schmeling [50, p.298], Barreira [6, p.5]):

### Question 1.2

Does every expanding repeller have an ergodic invariant measure of full dimension?

In this paper we will prove that the answer to Question 1.2 is negative by constructing a piecewise affine expanding repeller topologically conjugate to the full shift whose Hausdorff dimension is strictly greater than its dynamical dimension. This expanding repeller will belong to a class of sets that we call “self-affine sponges” (not all of which are expanding repellers), and we develop tools for calculating the Hausdorff and dynamical dimensions of self-affine sponges more generally. This makes our paper an extension of several known results about self-affine sponges [3, 10, 26, 28, 34], though in all previously studied cases, the Hausdorff and dynamical dimensions have turned out to be equal. We also note that self-affine sponges are a subclass of the more general class of self-affine sets, and that it is known that almost every self-affine set (with respect to a certain measure on the space of perturbations of a given self-affine set) has an ergodic invariant measure of full dimension [25]. However, self-affine sponges do not represent typical instances of self-affine sets and so this result does not contradict our theorems. Nevertheless, we show that our counterexamples represent a non-negligible set of self-affine sponges (in the sense of containing a nonempty open subset of the parameter space); see Theorem 2.9.

Previous approaches to Question 1.2 have involved using the thermodynamic formalism to compute the Hausdorff dimension of the repeller and then comparing with the dimensions of the invariant measures calculated using the Volume Lemma or its generalization, the Ledrappier–Young dimension formula [29, Corollary D$'$]. When it works, this strategy generally shows that the Hausdorff and dynamical dimensions of a repeller are equal. By contrast, we still use the Ledrappier–Young formula to calculate the dimension of invariant measures, but our strategy to calculate the dimension of the repeller is to pay more attention to the *non-invariant* measures. Indeed, we write the Hausdorff dimension of a self-affine sponge as the supremum of the Hausdorff dimensions of certain particularly nice non-invariant measures that we call “pseudo-Bernoulli” measures (see Definition 2.10), which are relatively homogeneous with respect to space, but whose behavior with respect to length scale varies in a periodic way. The dimension of these measures turns out to be calculable via an appropriate analogue of the Ledrappier–Young formula, which is how we show that it is sometimes larger than the dimension of any invariant measure.

## Main results

### Qualitative results

#### Definition 2.1

Fix $d \ge 1$, and let $D = \{1,\ldots ,d\}$. For each $i \in D$, let $A_i$ be a finite index set, and let $\Phi _i = (\phi _{i,a})_{a\in A_i}$ be a finite collection of contracting similarities of [0, 1], called the *base IFS in coordinate* *i*. (Here IFS is short for *iterated function system*.) Let $A = \prod _{i\in D} A_i$, and for each ${\mathbf {a}} = (a_1,\ldots ,a_d) \in A$, consider the contracting affine map $\phi _{{\mathbf {a}}} : [0,1]^d \rightarrow [0,1]^d$ defined by the formula

$$\begin{aligned} \phi _{{\mathbf {a}}}(x_1,\ldots ,x_d) = (\phi _{{\mathbf {a}},1}(x_1),\ldots ,\phi _{{\mathbf {a}},d}(x_d)), \end{aligned}$$

where $\phi _{{\mathbf {a}},i}$ is shorthand for $\phi _{i,a_i}$ in the formula above, as well as elsewhere. Geometrically, $\phi _{\mathbf {a}}$ can be thought of as corresponding to the rectangle

$$\begin{aligned} \phi _{\mathbf {a}}([0,1]^d) = \prod _{i\in D} \phi _{{\mathbf {a}},i}([0,1]) \subset [0,1]^d . \end{aligned}$$

Given $E \subset A$, we call the collection $\Phi {\, \mathop {=}\limits ^{\mathrm {def}}\, }(\phi _{\mathbf {a}})_{{\mathbf {a}}\in E}$ a *diagonal IFS*. The *coding map* of $\Phi$ is the map $\pi :E^\mathbb {N}\rightarrow [0,1]^d$ defined by the formula

$$\begin{aligned} \pi (\omega ) = \lim _{n\rightarrow \infty } \phi _{\omega \upharpoonleft n}(\mathbf {0}), \end{aligned}$$

where $\phi _{\omega \upharpoonleft n} {\, \mathop {=}\limits ^{\mathrm {def}}\, }\phi _{\omega _1}\circ \cdots \circ \phi _{\omega _n}$. Finally, the *limit set* of $\Phi$ is the set $\Lambda _\Phi {\, \mathop {=}\limits ^{\mathrm {def}}\, }\pi (E^\mathbb {N})$. We call the limit set of a diagonal IFS a *self-affine sponge*. It is a special case of the more general notion of an *self-affine set*, see e.g. [16].

#### Remark

This definition excludes some sets that it is also natural to call “sponges”, namely the limit sets of affine iterated function systems whose contractions preserve the class of coordinate-parallel rectangles, see e.g. [19]. The linear parts of such contractions are matrices that can be written as the composition of a permutation matrix and a diagonal matrix. Self-affine sets resulting from these “coordinate-permuting IFSes” are significantly more technical to deal with, so for simplicity we restrict ourselves to the case of sponges coming from diagonal IFSes.

When $d = 2$, self-affine sponges are called *self-affine carpets*, and have been studied in detail. Their Hausdorff dimensions were computed by Bedford [10], McMullen [34], Lalley and Gatzouras [28], and Barański [3], assuming that various conditions are satisfied. Since we will be interested in the higher-dimensional versions of these conditions, we define them now:

**Fig. 1:**
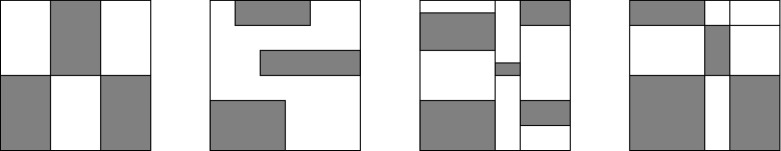
Generating templates for a Sierpiński carpet (*left*), carpets satisfying the coordinate ordering condition (*two middle pictures*), and a Barański carpet (*right*). Each picture defines a diagonal IFS: each *shaded region* corresponds to an affine contraction that sends the entire unit square to that *shaded region*. The *right middle picture* satisfies an additional disjointness condition which makes it a *Lalley–Gatzouras carpet*; cf. Definition 3.6

#### Definition 2.2

*(Cf. Fig.* 1 *)* Let $\Lambda _\Phi$ be a self-affine sponge defined by a diagonal IFS $\Phi$.

- We say that $\Phi$ or $\Lambda _\Phi$ is *Sierpiński* if the base IFSes are of the form
  $$\begin{aligned} \Phi _i&= (\phi _{i,a})_{0 \le a \le m_i - 1},&\phi _{i,a}(x)&= \frac{a + x}{m_i} \end{aligned}$$
  for some distinct integers $m_1,\ldots ,m_d \ge 2$.
- We say that $\Phi$ or $\Lambda _\Phi$ satisfies the *coordinate ordering condition* if there exists a permutation $\sigma$ of *D* such that for all ${\mathbf {a}}\in E$, we have
  $$\begin{aligned} |\phi _{{\mathbf {a}},\sigma (1)}'|> \cdots > |\phi _{{\mathbf {a}},\sigma (d)}'|. \end{aligned}$$
- We say that $\Phi$ or $\Lambda _\Phi$ is *Barański* (resp. *strongly Barański*) if the base IFSes all satisfy the open set condition (resp. the strong separation condition) with respect to the interval $\mathbb {I}= (0,1)$ (resp. $\mathbb {I}= [0,1]$), i.e. for all $i\in D$, the collection
  $$\begin{aligned} \big (\phi _{i,a}(\mathbb {I})\big )_{a\in A_i} \end{aligned}$$
  is disjoint.

Notice that every Sierpiński sponge satisfies the coordinate ordering condition and is also Barański. Bedford [10] and McMullen [34] independently computed the Hausdorff dimension of Sierpiński carpets, and consequently these carpets are sometimes known as *Bedford–McMullen carpets*. Barański computed the Hausdorff dimension of what we call Barański carpets [3].^1^ On the other hand, the coordinate ordering condition, which can be thought of as guaranteeing a “clear separation of Lyapunov directions”, cf. [7, p.643], is a higher-dimensional generalization of one of the assumptions of Lalley and Gatzouras [28]. Their other assumption is a disjointness condition [28, p.534] that is slightly weaker than the Barański condition. The higher-dimensional analogue of the disjointness condition is somewhat technical to state, so we defer its definition until Sect. 3.

#### Observation 2.3

Let $\Lambda _\Phi$ be a strongly Barański sponge. Then the coding map $\pi :E^\mathbb {N}\rightarrow \Lambda _\Phi$ is a homeomorphism. It follows that there is a unique map $f:\Lambda _\Phi \rightarrow \Lambda _\Phi$ such that $f\circ \pi = \pi \circ \sigma$, where $\sigma :E^\mathbb {N}\rightarrow E^\mathbb {N}$ is the shift map. In fact, the dynamical system $f:\Lambda _\Phi \rightarrow \Lambda _\Phi$ is a piecewise affine expanding repeller: for all ${\mathbf {a}}\in E$, we have $f = \phi _{\mathbf {a}}^{-1}$ on $\phi _{\mathbf {a}}(\Lambda _\Phi )$.

In [3, 10, 28, 34], a relation was established between the Hausdorff dimension of a self-affine carpet $\Lambda _\Phi$ and the Hausdorff dimension of the Bernoulli measures on $\Lambda _\Phi$. Here, a *Bernoulli measure* is a measure of the form

$$\begin{aligned} \nu _\mathbf {p}= \pi _*[\mathbf {p}^\mathbb {N}], \end{aligned}$$

where $\mathbf {p}$ is a probability measure on *E*, and $\pi _*[\mu ]$ denotes the pushforward of a measure $\mu$ under the coding map $\pi$. In what follows, we let $\mathcal P$ denote the space of probability measures on *E*.

#### Theorem 2.4

([3], special cases [10, 28, 34]) Let $\Lambda _\Phi$ be a Barański carpet (i.e. a two-dimensional Barański sponge). Then the Hausdorff dimension of $\Lambda _\Phi$ is equal to the supremum of the Hausdorff dimensions of the Bernoulli measures on $\Lambda _\Phi$, i.e.

2.1 $$\begin{aligned} {\dim _H}(\Phi ) = \sup _{\mathbf {p}\in \mathcal P} {\dim _H}(\nu _\mathbf {p}), \end{aligned}$$

where ${\dim _H}(\Phi )$ denotes the Hausdorff dimension of $\Lambda _\Phi$.

It is natural to ask whether Theorem 2.4 can be generalized to higher dimensions. This question was answered by Kenyon and Peres [26] in the case of Sierpiński sponges:

#### Theorem 2.5

([26, Theorem 1.2], special cases [10, 34]) The formula (2.1) holds for Sierpiński sponges (in all dimensions).

These results might lead one to conjecture that the formula (2.1) holds for all Barański sponges, or at least all Barański sponges satisfying the coordinate ordering condition. If that fails, one might still conjecture that the Hausdorff dimension of a Barański sponge is attained by some ergodic invariant measure, even if that measure is not a Bernoulli measure. For example, Neunhäuserer showed that the formula (2.1) fails for a certain class of non-Barański self-affine carpets [35, Theorem 2.2], but later it was shown that these carpets do in fact have ergodic invariant measures of full dimension [18, Theorem 2.15]. Similar examples appear in the realms of conformal iterated function systems satisfying the open set condition [24, 31, 32], affine iterated function systems with randomized translational parts [9, 25], and certain non-conformal non-affine iterated function systems [45], though in these settings, it was not expected that the measure of full dimension would be a Bernoulli measure. This leads to the following definition:

#### Definition 2.6

The *dynamical dimension* of a self-affine sponge $\Lambda _\Phi$ is the number

$$\begin{aligned} {\dim _D}(\Phi ) {\, \mathop {=}\limits ^{\mathrm {def}}\, }\sup _\mu \{{\dim _H}(\pi _*[\mu ])\}, \end{aligned}$$

where the supremum is taken over all probability measures $\mu$ on $E^\mathbb {N}$ that are invariant under the shift map.

It turns out that this definition does not help at getting larger dimensions:

#### Theorem 2.7

The dynamical dimension of a Barański sponge $\Lambda _\Phi$ is equal to the supremum of the Hausdorff dimensions of its Bernoulli measures, i.e.

2.2 $$\begin{aligned} {\dim _D}(\Phi ) = \sup _{\mathbf {p}\in \mathcal P} {\dim _H}(\nu _\mathbf {p}). \end{aligned}$$

The question remains whether the dynamical dimension is equal to the Hausdorff dimension of $\Lambda _\Phi$. It follows directly from the definition that

$$\begin{aligned} {\dim _H}(\Phi ) \ge {\dim _D}(\Phi ). \end{aligned}$$

The main result of this paper is that this inequality is sometimes strict:

#### Theorem 2.8

(Existence of sponges with a dimension gap) For all $d \ge 3$, there exists a strongly Barański sponge $\Lambda _\Phi \subset [0,1]^d$ satisfying the coordinate ordering condition such that

$$\begin{aligned} {\dim _H}(\Phi ) > {\dim _D}(\Phi ). \end{aligned}$$

Since the sponge $\Lambda _\Phi$ appearing in this theorem is strongly Barański, there exists a piecewise affine expanding repeller $f:\Lambda _\Phi \rightarrow \Lambda _\Phi$ such that $f\circ \pi = \pi \circ \sigma$, where $\sigma : E^\mathbb {N}\rightarrow E^\mathbb {N}$ is the shift map (cf. Observation 2.3). Thus, Theorem 2.8 shows that the answer to Question 1.2 is negative.

The contrast between Theorems 2.4 and 2.8 shows that the behavior of self-affine sponges is radically different in the two-dimensional and three-dimensional settings. See Remark 7.3 for some ideas about the cause of this difference.

A natural follow-up question is how common sponges with a dimension gap are. One way to measure this is to ask whether they represent a positive measure subset of the parameter space. We answer this question affirmatively by showing that dimension gaps are stable under perturbations: any Barański sponge whose defining IFS is sufficiently close to the defining IFS of a Barański sponge with a dimension gap also has a dimension gap. Equivalently, the class of Barański IFSes whose limit sets have a dimension gap is an open subset of the parameter space. This is an immediate corollary of the following theorem:

#### Theorem 2.9

The functions

2.3 $$\begin{aligned} \Phi&\mapsto {\dim _H}(\Phi ),&\Phi&\mapsto {\dim _D}(\Phi ) \end{aligned}$$

are continuous on the space of Barański IFSes.

#### Remark

It is not too hard to modify the proof of Theorem 2.9 to get a stronger result: the functions (2.3) are computable in the sense of computable analysis (see [54] for an introduction). This means that there is an algorithm that outputs arbitrarily accurate approximations of ${\dim _H}(\Phi )$ and ${\dim _D}(\Phi )$, given as input a sequence of approximations of $\Phi$. Every computable function is continuous [54, Theorem 4.3.1]; the converse is not true, since there are only countably many computable functions.

### Computational results

The strategy of the proof of Theorem 2.8 is to come up with general formulas for the Hausdorff and dynamical dimensions of a Barański sponge, and then to compare them in a concrete example. For example, Theorem 2.7 gives a way to compute the dynamical dimension once the dimensions of the Bernoulli measures are known. To get a similar result for the Hausdorff dimension, we introduce a new class of measures which we call “pseudo-Bernoulli”. These measures are not invariant, since if they were then their dimension could be no bigger than the dynamical dimension.

#### Definition 2.10

Recall that $\mathcal P$ denotes the space of probability measures on *E*, the alphabet of the IFS. Given $\lambda > 1$, we call a function $\mathbf {r}:(0,\infty )\rightarrow \mathcal P$ *exponentially* $\lambda$-*periodic* if for all $b > 0$, we have $\mathbf {r}_{\lambda b} = \mathbf {r}_b$. Here we denote the value of $\mathbf {r}$ at the argument *b* by $\mathbf {r}_b$ instead of $\mathbf {r}(b)$. We call $\mathbf {r}$ *exponentially* 1-*periodic* if it is constant. (The advantange of this definition is that the uniform limit of exponentially $\lambda$-periodic continuous functions as $\lambda \searrow 1$ is exponentially 1-periodic.) The class of exponentially $\lambda$-periodic continuous functions will be denoted $\mathcal R_\lambda$, and the union will be denoted $\mathcal R= \bigcup _{\lambda \ge 1} \mathcal R_\lambda$. Elements of $\mathcal R$ will be called *cycles on* *E*. Finally, a *pseudo-Bernoulli* measure is a measure of the form $\nu _\mathbf {r}{\, \mathop {=}\limits ^{\mathrm {def}}\, }\pi _*[\mu _\mathbf {r}]$, where $\mathbf {r}\in \mathcal R$, and

2.4 $$\begin{aligned} \mu _\mathbf {r}{\, \mathop {=}\limits ^{\mathrm {def}}\, }\prod _{n\in \mathbb {N}} \mathbf {r}_n \end{aligned}$$

is a probability measure on $E^\mathbb {N}$.

The following theorem subsumes Theorems 2.4 and 2.5 as special cases, see Sect. 7 for details. The techniques we use to prove it are similar to the techniques originally used to prove Theorems 2.4 and 2.5.

#### Theorem 2.11

The Hausdorff dimension of a Barański sponge $\Lambda _\Phi$ is equal to the supremum of the Hausdorff dimensions of its pseudo-Bernoulli measures, i.e.

2.5 $$\begin{aligned} {\dim _H}(\Phi ) = \sup _{\mathbf {r}\in \mathcal R} {\dim _H}(\nu _\mathbf {r}). \end{aligned}$$

#### Remark

The inequality ${\dim _H}(\Phi ) \ge \sup _{\mathbf {r}\in \mathcal R} {\dim _H}(\nu _\mathbf {r})$, which forms the easy direction of Theorem 2.11, is all that is needed in the proof of Theorem 2.8. However, the proof of Theorem 2.11 provides some motivation for why it is appropriate to consider measures of the form $\nu _\mathbf {r}$ in the proof of Theorem 2.8. Indeed, proving Theorem 2.11 is what caused the authors to start paying attention to the class of pseudo-Bernoulli measures.

Of course, Theorem 2.11 raises the question of how to compute the Hausdorff dimension of a pseudo-Bernoulli measure $\nu _\mathbf {r}$. Similarly, Theorem 2.7 raises the (easier) question of how to compute the Hausdorff dimension of a Bernoulli measure $\nu _\mathbf {p}$—which is answered by a Ledrappier–Young type formula (cf. (2.13)). In fact, the latter question can be viewed as a special case of the former, since every Bernoulli measure is also a pseudo-Bernoulli measure. As a matter of notation, if $\mathbf {p}\in \mathcal P$, then we let $\mathbf {p}$ also denote the constant cycle $b\mapsto \mathbf {p}_b = \mathbf {p}$, so that we can think of $\mathcal P$ as being equal to $\mathcal R_1 \subset \mathcal R$. Note that the notation $\nu _\mathbf {p}$ means the same thing whether we interpret it as referring to the Bernoulli measure corresponding to $\mathbf {p}\in \mathcal P$, or the pseudo-Bernoulli measure corresponding to the constant cycle $\mathbf {p}\in \mathcal R_1$.

To compute the Hausdorff dimension of pseudo-Bernoulli measures, we need to introduce some more notation and definitions:

#### Notation 2.12

For each $\mathbf {r}\in \mathcal R$ and $B > 0$, we let

2.6 $$\begin{aligned} \mathbf {R}_B&= \int _0^B \mathbf {r}_b\;\mathrm {d}b,&{{\widehat{\mathbf {R}}}}_B&= B^{-1} \mathbf {R}_B \in \mathcal P. \end{aligned}$$

Note that if $\mathbf {r}$ is exponentially $\lambda$-periodic, then so is ${\widehat{\mathbf {R}}}$. We will use a similar convention with other letters in place of $\mathbf {r}$; for example, if $\mathbf {p}\in \mathcal P$ then we write $\mathbf {P}_B = \int _0^B \mathbf {p}_b \;\mathrm {d}b = \int _0^B \mathbf {p}\;\mathrm {d}b = B\mathbf {p}$ and ${\widehat{\mathbf {P}}}_B = B^{-1} \mathbf {P}_B = \mathbf {p}$.

#### Definition 2.13

Given $\mathbf {p}\in \mathcal P$ and $i\in D$, the *i* *th Lyapunov exponent* 2 *of* $\mathbf {p}$ is the number

$$\begin{aligned} \chi _i(\mathbf {p}) {\, \mathop {=}\limits ^{\mathrm {def}}\, }-\int \log |\phi _{{\mathbf {a}},i}'| \;\mathrm {d}\mathbf {p}({\mathbf {a}}). \end{aligned}$$

Note that this definition makes sense even if the total mass of $\mathbf {p}$ is not 1, and we will use it sometimes in this more general sense. Given a coordinate set $I \subset D$, the *entropy of* *I* *with respect to* $\mathbf {p}$ is the number

$$\begin{aligned} h_I(\mathbf {p}) = h(I;\mathbf {p}) {\, \mathop {=}\limits ^{\mathrm {def}}\, }-\int \log \mathbf {p}([{\mathbf {a}}]_I) \;\mathrm {d}\mathbf {p}({\mathbf {a}}), \end{aligned}$$

where

2.7 $$\begin{aligned}{}[{\mathbf {a}}]_I = \{\mathbf {b}\in E : a_i = b_i \;\;\forall i \in I\}. \end{aligned}$$

Note that $[{\mathbf {a}}]_D = \{{\mathbf {a}}\}$ and $[{\mathbf {a}}]_{\emptyset }= E$.

Finally, given $I \subset I' \subset D$, then *conditional entropy of* $I'$ *relative to* *I* *with respect to* $\mathbf {p}$ is the number

$$\begin{aligned} h(I'\upharpoonleft I;\mathbf {p}) {\, \mathop {=}\limits ^{\mathrm {def}}\, }h(I';\mathbf {p}) - h(I;\mathbf {p}) = \int \log \frac{\mathbf {p}([{\mathbf {a}}]_I)}{\mathbf {p}([{\mathbf {a}}]_{I'})} \;\mathrm {d}\mathbf {p}({\mathbf {a}}). \end{aligned}$$

#### Definition 2.14

Given $\mathbf {r}\in \mathcal R$, we let $E_\mathbf {r}= \{{\mathbf {a}}\in E : \mathbf {r}_b({\mathbf {a}})> 0 \text { for some } b > 0\}$. We say that $\mathbf {r}$ is *nondegenerate* if the set $\{b> 0 : \mathbf {r}_b({\mathbf {a}}) > 0 \text { for all } {\mathbf {a}}\in E_\mathbf {r}\}$ is dense in $(0,\infty )$, and we denote the space of nondegenerate cycles by $\mathcal R^*$. We also write $\mathcal R_\lambda ^* = \mathcal R_\lambda \cap \mathcal R^*$.

Note that every measure is nondegenerate when considered as a constant cycle.

#### Theorem 2.15

Let $\Lambda _\Phi$ be a Barański sponge. Then for all $\lambda \ge 1$ and $\mathbf {r}\in \mathcal R_\lambda ^*$, the dimension ${\dim _H}(\nu _\mathbf {r})$ can be computed by the formula

2.8 $$\begin{aligned} {\dim _H}(\nu _\mathbf {r}) = \delta (\mathbf {r}) {\, \mathop {=}\limits ^{\mathrm {def}}\, }\inf _{B \in [1,\lambda ]} \delta (\mathbf {r},B), \end{aligned}$$

where for each $B > 0$,

2.9 $$\begin{aligned} \delta (\mathbf {r},B) {\, \mathop {=}\limits ^{\mathrm {def}}\, }\frac{1}{B} \int _0^\infty h(\{i\in D : b \le B_i\};\mathbf {r}_b) \;\mathrm {d}b, \end{aligned}$$

where the numbers $B_1,\ldots ,B_d > 0$ are chosen so that

2.10 $$\begin{aligned} B = \int _0^{B_i} \chi _i(\mathbf {r}_b) \;\mathrm {d}b = \chi _i(\mathbf {R}_{B_i}). \end{aligned}$$

If $\mathbf {r}\in \mathcal R_\lambda {\setminus }\mathcal R_\lambda ^*$, then ${\dim _H}(\nu _\mathbf {r}) \le \delta (\mathbf {r})$. The terms ${\dim _H}(\nu _\mathbf {r})$ $(\mathbf {r}\in \mathcal R_\lambda {\setminus }\mathcal R_\lambda ^*)$ do not contribute to the supremum in (2.5).

In particular, for all $\mathbf {p}\in \mathcal P$, the dimension ${\dim _H}(\nu _\mathbf {p})$ can be computed by the formula

2.11 $$\begin{aligned} {\dim _H}(\nu _\mathbf {p}) = \delta (\mathbf {p}) {\, \mathop {=}\limits ^{\mathrm {def}}\, }\int _0^\infty h(\{i\in D : b \le 1/\chi _i(\mathbf {p})\};\mathbf {p}) \;\mathrm {d}b. \end{aligned}$$

We remark that the map $B \mapsto \delta (\mathbf {r},B)$ is exponentially $\lambda$-periodic, so that the infimum in (2.8) would be the same if it was taken over all $B > 0$ rather than only over $B \in [1,\lambda ]$. We also remark on the geometric meaning of the quantities $B_1,\ldots ,B_d$: if $\omega \in E^\mathbb {N}$ is a $\mu _\mathbf {r}$-typical point and $\rho = e^{-B}$, then $B_i$ is approximately the number of coordinates of $\omega$ that must be known before the *i*th coordinate of $\pi (\omega )$ can be computed with accuracy $\rho$. Thus the numbers $B_1,\ldots ,B_d$ are useful at estimating the $\nu _\mathbf {r}$-measure of the ball $B(\pi (\omega ),\rho )$. For a more rigorous presentation of this idea, see the proof of Theorem 2.15.

Formulas (2.9) and (2.11) share a particularly nice feature, viz. their validity does not depend on the ordering of the numbers $B_1,\ldots ,B_d$ (in the case of (2.9)) or of the Lyapunov exponents $\chi _1(\mathbf {p}),\ldots ,\chi _d(\mathbf {p})$ (in the case of (2.11)). However, it is sometimes more useful to have versions of these formulas that do depend on the orderings of these numbers. For convenience, for all $i = 0,\ldots ,d$ we write

$$\begin{aligned} I_{\le i} = \{1,\ldots ,i\}, \end{aligned}$$

so that in particular $I_{\le 0} = \emptyset$ and $I_{\le d} = D$.

#### Proposition 2.16

If $B_1 \ge \cdots \ge B_d$ for some $\mathbf {r}\in \mathcal R$ and $B > 0$, then

2.12 $$\begin{aligned} \delta (\mathbf {r},B) = \sum _{i\in D} \frac{\int _0^{B_i} h(I_{\le i} \upharpoonleft I_{\le i - 1};\mathbf {r}_b) \;\mathrm {d}b}{\int _0^{B_i} \chi _i(\mathbf {r}_b)\;\mathrm {d}b} \le \sum _{i\in D} \frac{h(I_{\le i} \upharpoonleft I_{\le i - 1};{\widehat{\mathbf {R}}}_{B_i})}{\chi _i({\widehat{\mathbf {R}}}_{B_i})}\cdot \end{aligned}$$

In particular, if $\chi _1(\mathbf {p}) \le \cdots \le \chi _d(\mathbf {p})$ for some $\mathbf {p}\in \mathcal P$, then

2.13 $$\begin{aligned} \delta (\mathbf {p}) = \sum _{i\in D} \frac{h(I_{\le i} \upharpoonleft I_{\le i - 1};\mathbf {p})}{\chi _i(\mathbf {p})}\cdot \end{aligned}$$

#### Remark

The formula (2.13) is a special case of a theorem of Feng and Hu [18, Theorem 2.11]. It can be viewed as an analogue of the well-known Ledrappier–Young formula for the Hausdorff dimension of the unstable leaves of an ergodic invariant measure of a diffeomorphism [29, Corollary D$'$]. In fact, (2.13) is close to being a special case of the “Ledrappier–Young formula for endomorphisms” [44, Theorem 2.8 and (19)], although there are formal difficulties with deducing one from the other.^3^ Since the formula (2.12) bears some resemblance to (2.13), it can be thought of as extending this Ledrappier–Young-type formula to certain non-invariant measures of a dynamical system.

We remark that the results of this section are the first in the literature to address dimension questions regarding self-affine sponges of dimension at least three, with the exception of various results regarding Sierpiński sponges [26, 36, 37]. This significant gap in the literature was recently posed as question by Fraser and Howroyd [20, Question 4.3], namely how to compute the Hausdorff dimension and the upper and lower Assouad and box dimensions of self-affine sponges. The results of this subsection can be seen as partially answering this broad question.

*Outline of the paper* In Sect. 3 we introduce a weakening of the Barański assumption that we will use in our proofs. In Sect. 4 we prove Theorem 2.15 and Proposition 2.16. In Sect. 5 we prove Theorems 2.7 and 2.11. In Sect. 6 we prove Theorem 2.9. In Sect. 7 we give new proofs of Theorems 2.4 and 2.5 using Theorem 2.11. We prove our main result, Theorem 2.8, in Sect. 8. Finally, in Sect. 9 we list a few open questions. The sections are mostly independent of each other, but they are ordered according to the dependencies between the proofs.

*Notation* For the reader’s convenience we summarize a list of commonly used symbols below:

**Table:** 

IFS	Iterated function system
*d*	Dimension of the ambient Euclidean space
*D*	$D {\, \mathop {=}\limits ^{\mathrm {def}}\, }\{1,\ldots ,d\}$
$A_i$	The alphabet of the base IFS $\Phi _i$
$\Phi _i$	The base IFS in coordinate *i*: $\Phi _i = (\phi _{i,a})_{a\in A_i}$
*A*	The full product alphabet: $A {\, \mathop {=}\limits ^{\mathrm {def}}\, }\prod _{i\in D} A_i$
*E*	The alphabet of the IFS: $E \subset A$
$\Phi$	The diagonal IFS used to define the self-affine sponge: $\Phi {\, \mathop {=}\limits ^{\mathrm {def}}\, }(\phi _{\mathbf {a}})_{{\mathbf {a}}\in E}$
$\pi :E^\mathbb {N}\rightarrow [0,1]^d$	The coding map of $\Phi$
$\phi _{\omega \upharpoonleft n}$	IFS contraction corresponding to the word $\omega \upharpoonleft n$: $\phi _{\omega \upharpoonleft n} {\, \mathop {=}\limits ^{\mathrm {def}}\, }\phi _{\omega _1}\circ \cdots \circ \phi _{\omega _n}$
$\Lambda _\Phi$	The limit set of $\Phi$: $\Lambda _\Phi {\, \mathop {=}\limits ^{\mathrm {def}}\, }\pi (E^\mathbb {N})$
$\sigma :E^\mathbb {N}\rightarrow E^\mathbb {N}$	The shift map
$\pi _*[\mu ]$	Pushforward of a measure $\mu$ under the coding map $\pi$
$\mathcal P$	The space of probability measures on the alphabet *E*
$\nu _\mathbf {p}$	Bernoulli measure: $\nu _\mathbf {p}= \pi _*[\mathbf {p}^\mathbb {N}]$ for some $\mathbf {p}\in \mathcal P$
${\dim _H}$	Hausdorff dimension
${\dim _D}$	Dynamical dimension, see Definition 2.6
$\mathcal R$	Exponentially periodic continuous $\mathcal P$-valued functions, see Definition 2.10
$\mathcal R_\lambda$	Exponentially $\lambda$-periodic continuous $\mathcal P$-valued functions
$\mathcal Q$	Countable dense subset of $\mathcal R$
$\mathbf {r}_b$	Value of $\mathbf {r}:(0,\infty )\rightarrow \mathcal P$ at $b\in (0,\infty )$
$\mu _\mathbf {r}$	$\mu _\mathbf {r}{\, \mathop {=}\limits ^{\mathrm {def}}\, }\prod _{n\in \mathbb {N}} \mathbf {r}_n$
$\nu _\mathbf {r}$	Pseudo-Bernoulli measure: $\nu _\mathbf {r}{\, \mathop {=}\limits ^{\mathrm {def}}\, }\pi _*[\mu _\mathbf {r}]$ for some $\mathbf {r}\in \mathcal R$
$\mathbf {R}_B$, $\mathbf {S}_B$ $\hbox {etc}^\mathrm{a}$.	$\mathbf {R}_B {\, \mathop {=}\limits ^{\mathrm {def}}\, }\int _0^B \mathbf {r}_b\;\mathrm {d}b$
${\widehat{\mathbf {R}}}_B$, $\widehat{\mathbf {S}}_B$ etc.	${\widehat{\mathbf {R}}}_B {\, \mathop {=}\limits ^{\mathrm {def}}\, }B^{-1} \mathbf {R}_B \in \mathcal P$
$\chi _i(\mathbf {p})$	*i*th Lyapunov exponent of $\mathbf {p}$, see Definition 2.13
$h_I(\mathbf {p}) \equiv h(I;\mathbf {p})$	Entropy of *I* with respect to $\mathbf {p}$ for a coordinate set $I \subset D$, see Definition 2.13
$h(I'\upharpoonleft I;\mathbf {p})$	Conditional entropy of $I'$ relative to *I* with respect to $\mathbf {p}$, see Definition 2.13
$[{\mathbf {a}}]_I$	$[{\mathbf {a}}]_I {\, \mathop {=}\limits ^{\mathrm {def}}\, }\{\mathbf {b}\in E : a_i = b_i \;\;\forall i \in I\}$
$I_{\le i}$	$I_{\le i} {\, \mathop {=}\limits ^{\mathrm {def}}\, }\{1,\ldots ,i\}$

**Table:** 

$\mathcal R^*$	Nondegenerate cycles on *E*, see Definition 2.14
$\mathcal R_\lambda ^*$	$\mathcal R_\lambda ^* {\, \mathop {=}\limits ^{\mathrm {def}}\, }\mathcal R_\lambda \cap \mathcal R^*$
$\delta (\mathbf {p})$	Formula for computing ${\dim _H}(\nu _\mathbf {p})$: $\delta (\mathbf {p}) {\, \mathop {=}\limits ^{\mathrm {def}}\, }\int _0^\infty h(\{i\in D : b \le 1/\chi _i(\mathbf {p})\};\mathbf {p}) \;\mathrm {d}b$
$B_i$	The unique solution to $B = \int _0^{B_i} \chi _i(\mathbf {r}_b) \;\mathrm {d}b = \chi _i(\mathbf {R}_{B_i})$
$\delta (\mathbf {r},B)$	$\delta (\mathbf {r},B) {\, \mathop {=}\limits ^{\mathrm {def}}\, }\frac{1}{B} \int _0^\infty h(\{i\in D : b \le B_i\};\mathbf {r}_b) \;\mathrm {d}b$
$\delta (\mathbf {r})$	Formula for computing ${\dim _H}(\nu _\mathbf {r})$: $\delta (\mathbf {r}) {\, \mathop {=}\limits ^{\mathrm {def}}\, }\inf _{B \in [1,\lambda ]} \delta (\mathbf {r},B)$, where $\lambda$ is the exponential period of $\mathbf {r}$
$I(\mathbf {p},x)$	$I(\mathbf {p},x) {\, \mathop {=}\limits ^{\mathrm {def}}\, }\{i\in D : \chi _i(\mathbf {p}) \le x\}$
$\underline{\mathrm{d}}(\mathbf {x},\mu )$	Lower pointwise dimension of $\mu$ at $\mathbf {x}$
$\delta _x$	Dirac point measure at *x*
$X_i(\omega \upharpoonleft N)$	$X_i(\omega \upharpoonleft N) {\, \mathop {=}\limits ^{\mathrm {def}}\, }-\log |\phi _{\omega \upharpoonleft N,i}'|$
$[\omega \upharpoonleft N]_I$	$[\omega \upharpoonleft N]_I {\, \mathop {=}\limits ^{\mathrm {def}}\, }\{\tau \in E^\mathbb {N}: \tau _n \in [\omega _n]_I \;\;\forall n \le N\}$
$B_\omega (N_1,\ldots ,N_d)$	$B_\omega (N_1,\ldots ,N_d) {\, \mathop {=}\limits ^{\mathrm {def}}\, }\bigcap _{i\in D} [\omega \upharpoonleft {N_i}]_{\{i\}}$
$\mathbf {A}\cdot \mathbf {B}$	product of matrices $\mathbf {A}$ and $\mathbf {B}$
$\langle \mathbf {v},\mathbf {w}\rangle$	scalar product of vectors $\mathbf {v}$ and $\mathbf {w}$
*J*	$J {\, \mathop {=}\limits ^{\mathrm {def}}\, }\{1,2,3\}$ is the index set for the sub-IFSes of our construction
$\Delta$	Probability measures on *J*
$\mathbf {u}$	$\mathbf {u}{\, \mathop {=}\limits ^{\mathrm {def}}\, }(1/3,1/3,1/3)$
$\mathbf {U}$	$\mathbf {U}{\, \mathop {=}\limits ^{\mathrm {def}}\, }[1,1,1]^T\cdot [1,1,1]$

${}^\mathrm{a}$Expressions such as $\mathbf {S}_B$ sometimes appear without a corresponding function $b\mapsto \mathbf {s}_b\in \mathcal P$, such as in the proof of Theorem 3.3. However, in these cases the map $B\mapsto \mathbf {S}_B$ is still an increasing map from $(0,\infty )$ to the space of measures on *E* such that $\mathbf {S}_B(E) = B$ for all $B > 0$

## Weaker projection conditions

In the theorems of the previous section, we always assumed that the self-affine sponge in question was Barański—i.e. that its base IFSes satisfied the open set condition. This assumption is not always necessary and can in some circumstances be replaced by a weaker assumption:

### Definition 3.1

Let $\Lambda _\Phi$ be a self-affine sponge, and let $I \subset D$ be a coordinate set. Let

$$\begin{aligned} \Phi _I = (\phi _{I,{\mathbf {a}}})_{{\mathbf {a}}\in \pi _I(E)}, \end{aligned}$$

where $\phi _{I,{\mathbf {a}}}:[0,1]^I\rightarrow [0,1]^I$ is defined by the formula

$$\begin{aligned} \phi _{I,{\mathbf {a}}}(\mathbf {x}) = \big (\phi _{{\mathbf {a}},i}(x_i)\big )_{i\in I} \end{aligned}$$

and $\pi _I:A\rightarrow A_I {\, \mathop {=}\limits ^{\mathrm {def}}\, }\prod _{i\in I} A_i$ is the projection map. We call *I* *good* if the IFS $\Phi _I$ satisfies the open set condition, i.e. if the collection

$$\begin{aligned} \big (\phi _{I,{\mathbf {a}}}(\mathbb {I}^I)\big )_{{\mathbf {a}}\in \pi _I(E)} \end{aligned}$$

is disjoint, where $\mathbb {I}= (0,1)$. Also, a measure $\mathbf {p}\in \mathcal P$ is called *good* if for every $x > 0$, the set

3.1 $$\begin{aligned} I(\mathbf {p},x) = \{i\in D : \chi _i(\mathbf {p}) \le x\} \end{aligned}$$

is good. Next, a cycle $\mathbf {r}\in \mathcal R$ is called *good* if the measures ${\widehat{\mathbf {R}}}_B$ $(B > 0)$ are all good. Note that $\mathbf {p}$ is good as a measure if and only if it is good as a constant cycle. Finally, a sponge $\Lambda _\Phi$ is *good* if all measures (and thus also all cycles) on *E* are good. Note that every Barański sponge is good, since all of its coordinate sets are good.

### Theorem 3.2

(Generalization of Theorem 2.15) Let $\Lambda _\Phi$ be an arbitrary self-affine sponge. Then for all $\mathbf {r}\in \mathcal R$, we have

$$\begin{aligned} {\dim _H}(\nu _\mathbf {r}) \le \delta (\mathbf {r}), \end{aligned}$$

with equality if $\mathbf {r}$ is good and nondegenerate. Here $\delta (\mathbf {r})$ is defined in the same way as in Theorem 2.15. In particular, for all $\mathbf {p}\in \mathcal P$, we have

$$\begin{aligned} {\dim _H}(\nu _\mathbf {p}) \le \delta (\mathbf {p}), \end{aligned}$$

with equality if $\mathbf {p}$ is good.

### Theorem 3.3

Let $\Lambda _\Phi$ be an arbitrary self-affine sponge. Then

3.2 $$\begin{aligned} \sup _{\begin{array}{c} \mathbf {r}\in \mathcal R\\ \text {good} \end{array}}\delta (\mathbf {r}) \le {\dim _H}(\Phi )&\le \sup _{\mathbf {r}\in \mathcal R}\delta (\mathbf {r}), \end{aligned}$$

3.3 $$\begin{aligned} \sup _{\begin{array}{c} \mathbf {p}\in \mathcal P\\ \text {good} \end{array}} \delta (\mathbf {p}) \le {\dim _D}(\Phi )&\le \sup _{\mathbf {p}\in \mathcal P} \delta (\mathbf {p}). \end{aligned}$$

### Corollary 3.4

(Generalization of Theorems 2.7 and 2.11) Let $\Lambda _\Phi$ be a good sponge. Then

$$\begin{aligned} {\dim _H}(\Phi )&= \sup _{\mathbf {r}\in \mathcal R}\delta (\mathbf {r}),&{\dim _D}(\Phi )&= \sup _{\mathbf {p}\in \mathcal P} \delta (\mathbf {p}). \end{aligned}$$

### Remark 3.5

In some cases, Theorem 3.3 can still be used to compute the Hausdorff and dynamical dimensions of a sponge $\Lambda _\Phi$ even if that sponge is not good. This is because as long as the supremum of $\delta$ is attained at a good measure (resp. good cycle), then the dynamical (resp. Hausdorff) dimension of $\Lambda _\Phi$ is equal to the dimension of this measure (resp. cycle), regardless of whether or not other measures (resp. cycles) are good.

Using the terminology of this section, we can also generalize the framework of Lalley and Gatzouras [28] to higher dimensions:

### Definition 3.6

A sponge $\Lambda _\Phi$ will be called *Lalley–Gatzouras* if it satisfies the coordinate ordering condition with respect to some permutation $\sigma$ of *D*, such that the sets $\sigma (I_{\le i})$ ($i\in D$) are all good. Equivalently, a sponge is Lalley–Gatzouras if it is good and satisfies the coordinate ordering condition.

We do not prove any theorems specifically about Lalley–Gatzouras sponges, since they do not seem to behave any differently from general good sponges. However, it is worth noting that since all Lalley–Gatzouras sponges are good, all our theorems about good sponges apply to them, so that we are truly generalizing the framework of [28] as well as the framework of [3]. We also note that the sponge of Theorem 2.8 is a Lalley–Gatzouras sponge, since it is a Barański sponge that satisfies the coordinate ordering condition.

## Dimensions of pseudo-Bernoulli measures

In this section we compute the Hausdorff dimension of pseudo-Bernoulli measures, proving Theorem 3.2 (which implies Theorem 2.15) and Proposition 2.16. Our main tool will be the Rogers–Taylor density theorem, a well-known formula for computing the Hausdorff dimension of a measure:

### Theorem 4.1

([46]) If $\mu$ is a probability measure on $\mathbb {R}^d$ and $S \subset \mathbb {R}^d$ is a set of positive $\mu$-measure, thenwhereis the lower pointwise dimension of $\mu$ at $\mathbf {x}$. In particular,


We prove Proposition 2.16 first, since it will be used in the proof of Theorem 3.2. We need a lemma, which will also be used in the proof of Theorem 2.8:

### Lemma 4.2

(Near-linearity of entropy) Let *J* be a finite set, let $(q_j)_{j\in J}$ be a probability vector, and let $(\mathbf {p}_j)_{j\in J}$ be a family of elements of $\mathcal P$. Then for all $I \subset I' \subset D$,

4.1 $$\begin{aligned} \sum _{j\in J} q_j h(I'\upharpoonleft I;\mathbf {p}_j) \le h\left( I'\upharpoonleft I ; \sum _{j\in J} q_j \mathbf {p}_j\right) \le \sum _{j\in J} q_j h(I'\upharpoonleft I;\mathbf {p}_j) + \log \#(J). \end{aligned}$$

### Proof

Let $\mathbf {p}$ be the probability measure on $J\times E$ given by the formula $\mathbf {p}= \sum _{j\in J} q_j \delta _j\times \mathbf {p}_j$, where $\delta _j$ denotes the Dirac point measure at *j*. Consider the partitions on $J\times E$ given by the formulas

$$\begin{aligned} \mathcal A&{\, \mathop {=}\limits ^{\mathrm {def}}\, }\{J\times [{\mathbf {a}}]_{I'} : {\mathbf {a}}\in E\},&\mathcal B&{\, \mathop {=}\limits ^{\mathrm {def}}\, }\{J\times [{\mathbf {a}}]_I : {\mathbf {a}}\in E\},&\mathcal C&{\, \mathop {=}\limits ^{\mathrm {def}}\, }\{\{j\}\times E : j\in J\}. \end{aligned}$$

Then (4.1) is equivalent to the inequalities

$$\begin{aligned} H_\mathbf {p}(\mathcal A\upharpoonleft \mathcal B\vee \mathcal C) \le H_\mathbf {p}(\mathcal A\upharpoonleft \mathcal B) \le H_\mathbf {p}(\mathcal A\upharpoonleft \mathcal B\vee \mathcal C) + \log \#(\mathcal C), \end{aligned}$$

where $H_\mathbf {p}(\cdot \upharpoonleft \cdot )$ denotes the standard conditional entropy of two partitions. These inequalities follow from well-known facts about entropy, see e.g. [43, Theorem 2.3.3(f)]. $\square$

### Corollary 4.3

Let *J* be a Borel measurable space, let $\mathbf {q}$ be a probability measure on *J*, and let $(\mathbf {p}_j)_{j\in J}$ be a family of elements of $\mathcal P$. Then for all $I \subset I' \subset D$,

$$\begin{aligned} \int h(I'\upharpoonleft I;\mathbf {p}_j) \;\mathrm {d}\mathbf {q}(j) \le h\left( I'\upharpoonleft I ; \int \mathbf {p}_j \;\mathrm {d}\mathbf {q}(j)\right) . \end{aligned}$$

### Proof

If $\mathcal A$ is a finite partition of *J*, then Lemma 4.2 shows that

$$\begin{aligned} \sum _{A\in \mathcal A} h\left( I'\upharpoonleft I ; \frac{1}{\mathbf {q}(A)} \int _A \mathbf {p}_j \;\mathrm {d}\mathbf {q}(j) \right) \mathbf {q}(A) \le h\left( I'\upharpoonleft I ; \int \mathbf {p}_j \;\mathrm {d}\mathbf {q}(j)\right) . \end{aligned}$$

Letting $\mathcal A$ tend to the partition of *J* into points completes the proof. $\square$

### Proof of Proposition 2.16

Write $B_{d + 1} = 0$, so that $B_1 \ge \cdots \ge B_{d + 1}$. Then

$$\begin{aligned} \{i\in D : b \le B_i\} = I_{\le j} \;\;\forall j = 1,\ldots ,d \;\;\forall b\in (B_{j + 1},B_j), \end{aligned}$$

and $\{i\in D : b \le B_i\} = \emptyset$ for all $b > B_1$. Thus

$$\begin{aligned} B\delta (\mathbf {r},B)&= \int h(\{i\in D : b \le B_i\};\mathbf {r}_b) \;\mathrm {d}b\\&= \sum _{i = 1}^d \int _{B_{i + 1}}^{B_i} h(I_{\le i};\mathbf {r}_b)\;\mathrm {d}b\\&= \sum _{i = 1}^d \int _0^{B_i} h(I_{\le i};\mathbf {r}_b)\;\mathrm {d}b - \sum _{i = 2}^{d + 1} \int _0^{B_i} h(I_{\le i - 1};\mathbf {r}_b)\;\mathrm {d}b\\&= \sum _{i = 1}^d \int _0^{B_i} h(I_{\le i} \upharpoonleft I_{\le i - 1};\mathbf {r}_b)\;\mathrm {d}b\\&\le \sum _{i = 1}^d B_i h(I_{\le i} \upharpoonleft I_{\le i - 1};{\widehat{\mathbf {R}}}_B). \quad \text {(by Corollary } 4.3\text {)} \end{aligned}$$

Dividing by *B* and then applying (2.10) yields (2.12). Considering the special case where $\mathbf {r}$ is constant yields (2.13). $\square$

### Proof of Theorem 3.2

For convenience, in this proof we use the max norm on $\mathbb {R}^d$. Fix $\mathbf {r}\in \mathcal R$, and let $\omega _1,\omega _2,\ldots$ be a sequence of *E*-valued independent random variables, such that the distribution of $\omega _n$ is $\mathbf {r}_n$. Then $\omega = \omega _1\omega _2\cdots$ is an $E^\mathbb {N}$-valued random variable with distribution $\mu _\mathbf {r}$. For each $i\in D$, consider the sequence of random variables

$$\begin{aligned} \big (-\log |\phi _{\omega _n,i}'|\big )_{n\in \mathbb {N}} \end{aligned}$$

and for each $I\subset D$, consider the sequence of random variables

$$\begin{aligned} \big (-\log \mathbf {r}_n([\omega _n]_I)\big )_{n\in \mathbb {N}} \end{aligned}$$

(cf. (2.7)). Each of these sequences is a sequence of independent random variables with uniformly bounded variance,^4^ so by [11, Corollary A.8]^5^ the law of large numbers holds for these sequences, i.e.

$$\begin{aligned} -\sum _{n = 1}^N \log |\phi _{\omega _n,i}'|&= \sum _{n = 1}^N \chi _i(\mathbf {r}_n) + o(N)\\ -\sum _{n = 1}^N \log \mathbf {r}_n([\omega _n]_I)&= \sum _{n = 1}^N h_I(\mathbf {r}_n) + o(N) \end{aligned}$$

almost surely. Moreover, since $\mathbf {r}\in \mathcal R$, we have

$$\begin{aligned} \sup _{\begin{array}{c} b,b' \ge B \\ |b - b'| \le 1 \end{array}} \Vert \mathbf {r}_{b'} - \mathbf {r}_b\Vert \xrightarrow [B\rightarrow \infty ]{} 0, \end{aligned}$$

where $\Vert \cdot \Vert$ is any norm on the space of measures of *E*. Since the functions $\chi _i$ ($i\in D$) and $h_I$ ($I\subset D$) are continuous, this implies that

$$\begin{aligned} \sum _{n = 1}^N \chi _i(\mathbf {r}_n)&= \int _0^N \chi _i(\mathbf {r}_b) \;\mathrm {d}b + o(N)\\ \sum _{n = 1}^N h_I(\mathbf {r}_n)&= \int _0^N h_I(\mathbf {r}_b) \;\mathrm {d}b + o(N). \end{aligned}$$

Now let us introduce the notation

$$\begin{aligned} X_i(\omega \upharpoonleft N)&= -\log |\phi _{\omega \upharpoonleft N,i}'|\\ [\omega \upharpoonleft N]_I&= \{\tau \in E^\mathbb {N}: \tau _n \in [\omega _n]_I \;\;\forall n \le N\}, \end{aligned}$$

so that

$$\begin{aligned} X_i(\omega \upharpoonleft N) = -\sum _{n = 1}^N \log |\phi _{\omega _n,i}'|&= \chi _i(\mathbf {R}_N) + o(N)\\ -\log \mu _\mathbf {r}([\omega \upharpoonleft N]_I) = -\sum _{n = 1}^N \log \mathbf {r}_n([\omega _n]_I)&= \int _0^N h_I(\mathbf {r}_b) \;\mathrm {d}b + o(N). \end{aligned}$$

For all $N_1,\ldots ,N_d \in \mathbb {N}$, write

4.2 $$\begin{aligned} B_\omega (N_1,\ldots ,N_d) {\, \mathop {=}\limits ^{\mathrm {def}}\, }\bigcap _{i\in D} [\omega \upharpoonleft {N_i}]_{\{i\}}, \end{aligned}$$

and note that

$$\begin{aligned} {{\mathrm{diam}}}\big (\pi \big (B_\omega (N_1,\ldots ,N_d)\big )\big ) \le \max _{i\in D} \exp (-X_i(\omega \upharpoonleft {N_i})) \end{aligned}$$

since we are using the max norm. Now let $\rho > 0$ be a small number, let $B = -\log (\rho )$, and let $B_1,\ldots ,B_d > 0$ be given by (2.10). Without loss of generality suppose that $B_1 \ge \cdots \ge B_d$.

We proceed to prove that ${\dim _H}(\nu _\mathbf {r}) \le \delta (\mathbf {r})$. Fix $\varepsilon > 0$, and for each $i\in D$ let $N_i = \lfloor (1 + \varepsilon ) B_i\rfloor$. Then if *B* is sufficiently large (depending on $\varepsilon$), then

$$\begin{aligned} X_i(\omega \upharpoonleft {N_i}) \ge \chi _i(\mathbf {R}_{B_i}) = B = -\log (\rho ) \;\;\forall i\in D, \end{aligned}$$

and thus

$$\begin{aligned} \pi \big (B_\omega (N_1,\ldots ,N_d)\big ) \subset B(\pi (\omega ),\rho ). \end{aligned}$$

So

$$\begin{aligned} -\log \nu _\mathbf {r}\big (B(\pi (\omega ),\rho )\big )&\le -\log \mu _\mathbf {r}\big (B_\omega (N_1,\ldots ,N_d)\big )\\&= -\sum _{n\in \mathbb {N}} \log \mathbf {r}_n([\omega _n]_{\{i\in D : n\le N_i\}}) \\&= -\sum _{i\in D} \sum _{n = N_{i + 1} + 1}^{N_i} \log \mathbf {r}_n([\omega _n]_{I_{\le i}}) \quad (\text {with } N_{d + 1} {\, \mathop {=}\limits ^{\mathrm {def}}\, }0)\\&= \sum _{i\in D} \int _{N_{i + 1}}^{N_i} h(I_{\le i};\mathbf {r}_b) \;\mathrm {d}b + o(N_i)\\&= \sum _{i\in D} \int _0^{N_i} h(I_{\le i}\upharpoonleft I_{\le i - 1};\mathbf {r}_b) \;\mathrm {d}b + o(B)\\&= \sum _{i\in D} \int _0^{B_i} h(I_{\le i}\upharpoonleft I_{\le i - 1};\mathbf {r}_b) \;\mathrm {d}b + O(\varepsilon B) + o(B) \\&= B[\delta (\mathbf {r},B) + O(\varepsilon ) + o(1)] \quad \text {(by Proposition 2.16)} \end{aligned}$$

and thus

$$\begin{aligned} \frac{\log \nu _\mathbf {r}\big (B(\pi (\omega ),\rho )\big )}{\log (\rho )} \le \delta (\mathbf {r},B) + O(\varepsilon ) + o(1). \end{aligned}$$

Letting $B\rightarrow \infty$ (i.e. $\rho \rightarrow 0$) and then $\varepsilon \rightarrow 0$, we get

$$\begin{aligned} \underline{\mathrm{d}}(\pi (\omega ),\nu _\mathbf {r}) \le \liminf _{B\rightarrow \infty } \delta (\mathbf {r},B), \end{aligned}$$

where $\underline{\mathrm{d}}$ is as in Theorem 4.1. But since $\mathbf {r}$ is exponentially periodic, so is $B\mapsto \delta (\mathbf {r},B)$, and thus

$$\begin{aligned} \liminf _{B\rightarrow \infty } \delta (\mathbf {r},B) = \inf _{B\in [1,\lambda ]} \delta (\mathbf {r},B) = \delta (\mathbf {r}). \end{aligned}$$

Combining with Theorem 4.1 proves that ${\dim _H}(\nu _\mathbf {r}) \le \delta (\mathbf {r})$.

Now suppose that $\mathbf {r}$ is good and nondegenerate, and we will show that ${\dim _H}(\nu _\mathbf {r}) \ge \delta (\mathbf {r})$. Without loss of generality assume that $E_\mathbf {r}= E$. Consider the numbers $N_i {\, \mathop {=}\limits ^{\mathrm {def}}\, }\lfloor (1 - \varepsilon ) B_i\rfloor$ ($i\in D$). We will show that

4.3 $$\begin{aligned} \pi ^{-1}\big (B(\pi (\omega ),\rho )\big ) \subset B_\omega (N_1,\ldots ,N_d) \text { for all } B \text { sufficiently large} \end{aligned}$$

almost surely. By the preceding calculations, this suffices to finish the proof.

We consider the auxiliary numbers $M_i {\, \mathop {=}\limits ^{\mathrm {def}}\, }\lfloor (1 - \varepsilon /2) B_i\rfloor$ ($i\in D$). We also let

$$\begin{aligned} \varepsilon _0&= \min _{i\in D} \min _{x\in \{0,1\}} \min _{\begin{array}{c} a\in A_i \\ x\notin \phi _{i,a}([0,1]) \end{array}} \mathrm{dist}\big (x,\phi _{i,a}([0,1])\big ),&C&= -\log (\varepsilon _0). \end{aligned}$$

If *B* is sufficiently large (depending on $\varepsilon$), then

4.4 $$\begin{aligned} X_i(\omega \upharpoonleft {M_i}) < \chi _i(\mathbf {R}_{B_i}) - C = B - C = -\log (\rho /\varepsilon _0) \;\;\forall i\in D. \end{aligned}$$

Now fix $i\in D$, and consider the sequence of random events

$$\begin{aligned} \big (E_n(i) {\, \mathop {=}\limits ^{\mathrm {def}}\, }\big [\phi _{\omega _n,i}\circ \phi _{\omega _{n + 1},i}([0,1]) \subset (0,1)\big ]\big )_{n\in \mathbb {N}}. \end{aligned}$$

These events are not independent, but the subsequences corresponding to even and odd indices are both sequences of independent events. So again by [11, Corollary 1.8 in the Appendix], we have

$$\begin{aligned} \#\{n \le N : E_n(i) \text { holds}\} = \sum _{n = 1}^N p_n + o(N), \end{aligned}$$

almost surely, where $p_n$ is the probability of $E_n$. In particular, for all $j\in D$

$$\begin{aligned} \#\{N_j< n < M_j : E_n(i) \text { holds}\} = \sum _{n = N_j}^{M_j} p_n + o(M_j). \end{aligned}$$

Letting

4.5 $$\begin{aligned} f(\mathbf {p}) = \mathbf {p}\times \mathbf {p}(\{({\mathbf {a}},\mathbf {b})\in E^2 : \phi _{{\mathbf {a}},i}\circ \phi _{\mathbf {b},i}([0,1]) \subset (0,1)\}), \end{aligned}$$

we have $p_n = f(\mathbf {r}_n) + o(1)$ and thus

$$\begin{aligned} \#\{N_j< n < M_j : E_n(i) \text { holds}\} = \int _{(1 - \varepsilon )B_j}^{(1 - \varepsilon /2)B_j} f(\mathbf {r}_b) \;\mathrm {d}b + o(B). \end{aligned}$$

Now without loss of generality suppose that $\phi _{{\mathbf {a}},i} \circ \phi _{\mathbf {b},i}([0,1]) \subset (0,1)$ for some ${\mathbf {a}},\mathbf {b}\in E$. (If not, then there exists $x \in \{0,1\}$ such that $\phi _{{\mathbf {a}},i}(x) = x$ for all ${\mathbf {a}}\in E$, in which case the coordinate *i* can be ignored since its value is constant over the entire sponge $\Lambda _\Phi$.) Then $f(\mathbf {p}) > 0$ for all $\mathbf {p}\in \mathcal P$ such that $\mathbf {p}({\mathbf {a}}) > 0$ for all ${\mathbf {a}}\in E = E_\mathbf {r}$. So since $\mathbf {r}$ is nondegenerate, we have

$$\begin{aligned} \int _{(1 - \varepsilon )B_i}^{(1 - \varepsilon /2)B_i} f(\mathbf {r}_b) \;\mathrm {d}b \ge \delta B \end{aligned}$$

for some $\delta > 0$ depending on $\varepsilon$. So we have

4.6 $$\begin{aligned} \{N_j< n < M_j : E_n(i) \text { holds}\} \ne \emptyset \end{aligned}$$

for all *B* sufficiently large (depending on $\varepsilon$).

Now fix $\tau \in E^\mathbb {N}$ such that $\pi (\tau ) \in B(\pi (\omega ),\rho )$, and we will show that $\tau \in B_\omega (N_1,\ldots ,N_d)$. Indeed, by contradiction, suppose that $\tau \notin [\omega \upharpoonleft {N_j}]_{\{j\}}$ for some $j\in D$, and let

$$\begin{aligned} I = I({\widehat{\mathbf {R}}}_{B_j},B/B_j) = \{i\in D : B_i \ge B_j\} \end{aligned}$$

(cf. (3.1)). Since $\mathbf {r}$ is good, so is *I*. Moreover, since $j\in I$, we have $\tau \notin [\omega \upharpoonleft {N_j}]_I$. Write $N = N_j$ and $M = M_j$. Then

$$\begin{aligned}&\rho \ge \mathrm{dist}(\pi _I(\omega ),\pi _I(\tau ))\\&\quad \ge \mathrm{dist}\big (\phi _{\omega \upharpoonleft M,I}([0,1]^I),\mathbb {R}^I{\setminus }\phi _{\omega \upharpoonleft N,I}((0,1)^I)\big )&\quad \text {(since }I \text {is good)}\\&\quad \ge \varepsilon _0 \min _{i\in I} \big |\phi _{\omega \upharpoonleft M,i}'\big |&\quad \text {(by } (4.6)\text {)}\\&\quad = \varepsilon _0 \exp \big (-\max _{i\in I} X_i(\omega \upharpoonleft {M_j})\big )\\&\quad \ge \varepsilon _0 \exp \big (-\max _{i\in I} X_i(\omega \upharpoonleft {M_i})\big ),&\text {(since } B_i \ge B_j \;\;\forall i\in I \text {)} \end{aligned}$$

which contradicts (4.4). This demonstrates (4.3), completing the proof. $\square$

## Hausdorff and dynamical dimensions of self-affine sponges

In this section we compute the Hausdorff and dynamical dimensions of a self-affine sponge by proving Theorem 3.3, which implies Theorems 2.7 and 2.11.

### Proof of Theorem 3.3

Let $\mathbf {r}\in \mathcal R$ be a good cycle. Fix $0< \varepsilon < 1$, and let

$$\begin{aligned} \mathbf {s}_b = (1 - \varepsilon )\mathbf {r}_{b^{1 - \varepsilon }} + \varepsilon {\widehat{\mathbf {R}}}_{b^{1 - \varepsilon }}, \end{aligned}$$

so that $\mathbf {S}_B = B^\varepsilon \mathbf {R}_{B^{1 - \varepsilon }}$. Since $\mathbf {r}$ is a good cycle, so is $\mathbf {s}$. For all ${\mathbf {a}}\in E_\mathbf {s}= E_\mathbf {r}$ and $b > 0$, we have ${\widehat{\mathbf {R}}}_{b^{1 - \varepsilon }}({\mathbf {a}}) > 0$ and thus $\mathbf {s}_b({\mathbf {a}}) > 0$, so $\mathbf {s}$ is nondegenerate. Thus by Theorem 3.2, we have

$$\begin{aligned} {\dim _H}(\Phi ) \ge {\dim _H}(\nu _\mathbf {s}) = \delta (\mathbf {s}) \xrightarrow [\varepsilon \rightarrow 0]{} \delta (\mathbf {r}). \end{aligned}$$

Taking the supremum over all good $\mathbf {r}\in \mathcal R$ proves the left-hand inequality of (3.2). On the other hand, the left-hand inequality of (3.3) is immediate from Theorem 3.2.

We will now prove the right-hand inequalities of (3.2) and (3.3). For each $\mathbf {r}\in \mathcal R$ and $\varepsilon > 0$, we let

$$\begin{aligned} S_{\mathbf {r},\varepsilon } = \big \{\mathbf {x}\in \Lambda _\Phi : \underline{\mathrm{d}}(\mathbf {x},\nu _\mathbf {r}) \le \delta (\mathbf {r}) + \varepsilon \big \}, \end{aligned}$$

where $\delta (\mathbf {r})$ denotes the right-hand side of (2.8). By Theorem 4.1, we have ${\dim _H}(S_{\mathbf {r},\varepsilon }) \le \delta (\mathbf {r}) + \varepsilon$. Now for each rational $\lambda \ge 1$ let $\mathcal Q_\lambda$ be a countable dense subset of $\mathcal R_\lambda$, and let $\mathcal Q= \bigcup _{1 \le \lambda \in \mathbb {Q}} \mathcal Q_\lambda$. Then since Hausdorff dimension is $\sigma$-stable, the sets

$$\begin{aligned} S_1&{\, \mathop {=}\limits ^{\mathrm {def}}\, }\bigcap _{\varepsilon> 0} \bigcup _{\mathbf {r}\in \mathcal Q} S_{\mathbf {r},\varepsilon }\\ S_2&{\, \mathop {=}\limits ^{\mathrm {def}}\, }\bigcap _{\varepsilon > 0} \bigcup _{\mathbf {p}\in \mathcal Q_1} S_{\mathbf {p},\varepsilon } \end{aligned}$$

satisfy

$$\begin{aligned} {\dim _H}(S_1)&\le \sup _{\mathbf {r}\in \mathcal Q} \delta (\mathbf {r}),\\ {\dim _H}(S_2)&\le \sup _{\mathbf {p}\in \mathcal Q_1} \delta (\mathbf {p}). \end{aligned}$$

To complete the proof, we need to show that

5.1 $$\begin{aligned} {\dim _H}(\Phi )&\le {\dim _H}(S_1), \end{aligned}$$

5.2 $$\begin{aligned} {\dim _D}(\Phi )&\le {\dim _H}(S_2). \end{aligned}$$

We will prove (5.1) first, since afterwards it will be easy to modify the proof to show (5.2). Fix $\omega \in E^\mathbb {N}$, and we will show that $\pi (\omega ) \in S_1$. For each $N\in \mathbb {N}$ let

5.3 $$\begin{aligned} \mathbf {P}_N&= \sum _{n = 1}^N \delta _{\omega _n},&{\widehat{\mathbf {P}}}_N&= \frac{1}{N} \mathbf {P}_N. \end{aligned}$$

If $\mathbf {p}$ is a signed measure on *E*, then we let

$$\begin{aligned} \Vert \mathbf {p}\Vert = \sum _{{\mathbf {a}}\in E} |\mathbf {p}({\mathbf {a}})|. \end{aligned}$$

### Claim 5.1

For all $C > 1$ and $\varepsilon > 0$, there exist $1 < \lambda \in \mathbb {Q}$ and $\mathbf {r}\in \mathcal Q_\lambda$ such that for all $B\in [1,\lambda ]$,

5.4 $$\begin{aligned} \liminf _{k\rightarrow \infty }\sup _{M\in [C^{-1} \lambda ^k B,C \lambda ^k B]} \Vert {\widehat{\mathbf {R}}}_M - {\widehat{\mathbf {P}}}_M\Vert \le \varepsilon . \end{aligned}$$

Moreover, $\mathbf {r}$ may be taken so that $\mathbf {r}_b \in \mathcal P^*$ for all $b > 0$, where

$$\begin{aligned} \mathcal P^* {\, \mathop {=}\limits ^{\mathrm {def}}\, }\{\mathbf {p}\in \mathcal P: \mathbf {p}({\mathbf {a}}) > 0 \;\;\forall {\mathbf {a}} \in E\}. \end{aligned}$$

### Proof

By compactness, there is a sequence of *N*s such that for all $B\in \mathbb {Q}^+$ we have

5.5 $$\begin{aligned} \frac{1}{N}\mathbf {P}_{N B} \dashrightarrow \mathbf {Q}_B, \end{aligned}$$

where $\dashrightarrow$ indicates convergence along this sequence. Since the map $\mathbb {Q}^+\ni B\mapsto \mathbf {Q}_B$ is increasing and uniformly continuous (in fact 1-Lipschitz), it can be extended to an increasing continuous map $\mathbb {R}^+ \ni B\mapsto \mathbf {Q}_B$. Note that $\mathbf {Q}_B(E) = B$ for all $B\in \mathbb {R}^+$. Write $\widehat{\mathbf {Q}}_B = B^{-1}\mathbf {Q}_B \in \mathcal P$.

Fix $0< \varepsilon _3< \varepsilon _2 < 1$ small to be determined. For each $t\in \mathbb {R}$ write $\mathbf {q}(t) = \widehat{\mathbf {Q}}_{\exp (t)}$. For all $t_2 > t_1$, we have

$$\begin{aligned}&\Vert \mathbf {q}(t_2) - \mathbf {q}(t_1)\Vert \\&\quad = \Vert e^{-t_2} \mathbf {Q}_{\exp (t_2)} - e^{-t_1} \mathbf {Q}_{\exp (t_1)}\Vert \\&\quad = \Vert e^{-t_2} (e^{t_1} {\mathbf {a}} + (e^{t_2} - e^{t_1}) \mathbf {b}) - e^{-t_1} (e^{t_1} {\mathbf {a}})\Vert&\text {(for some } {\mathbf {a}},\mathbf {b}\in \mathcal P\text {)}\\&\quad = \Vert e^{-t_2} (e^{t_2} - e^{t_1}) (\mathbf {b}- {\mathbf {a}})\Vert \\&\quad \le 2 e^{-t_2} (e^{t_2} - e^{t_1}) \le 2 (t_2 - t_1),&\text {(since } \Vert {\mathbf {a}}\Vert = \Vert \mathbf {b}\Vert = 1\text {)} \end{aligned}$$

i.e. $\mathbf {q}$ is 2-Lipschitz. By the Arzela–Ascoli theorem the collection of all 2-Lipschitz maps from $\mathbb {R}$ to $\mathcal P$ is compact in the topology of locally uniform convergence. Since the translated paths $t\mapsto \mathbf {q}(T + t)$ ($T\in \mathbb {R}$) are members of this collection, it follows that there exist $T_1,T_2\in \mathbb {R}$ with $\rho _1 {\, \mathop {=}\limits ^{\mathrm {def}}\, }T_2 - T_1 \ge \log (C)$, such that for all $t\in [-\log (C),\log (C)]$, $\Vert \mathbf {q}(T_2 + t) - \mathbf {q}(T_1 + t)\Vert \le \varepsilon _3$. Let $A_1 = \exp (T_1)$, $A_2 = \exp (T_2)$, and $\lambda _1 = A_2/A_1 = \exp (\rho _1) \ge C$. Then

5.6 $$\begin{aligned} \text {for all } B\in [C^{-1},C], \text { we have } \Vert \widehat{\mathbf {Q}}_{A_2 B} - \widehat{\mathbf {Q}}_{A_1 B}\Vert \le \varepsilon _3. \end{aligned}$$

Now for each $B\in [A_1,A_2]$, let $\mathbf {S}_B = (1 - \varepsilon _2)\mathbf {Q}_B + \varepsilon _2 B\mathbf {u}$ and $\widehat{\mathbf {S}}_B = B^{-1} \mathbf {S}_B = (1 - \varepsilon _2)\widehat{\mathbf {Q}}_B + \varepsilon _2 \mathbf {u}$, where $\mathbf {u}\in \mathcal P$ is the normalized uniform measure on *E*. (We will later define $\mathbf {S}_B$ for $B\notin [A_1,A_2]$ as well, but not with this formula.) Let $\delta = \#(E) \varepsilon _3/\varepsilon _2 > 0$. Then

$$\begin{aligned} (1 + \delta )\widehat{\mathbf {S}}_{A_1} - \widehat{\mathbf {S}}_{A_2}&= (1 - \varepsilon _2)((1 + \delta )\widehat{\mathbf {Q}}_{A_1} - \widehat{\mathbf {Q}}_{A_2}) + \delta \varepsilon _2 \mathbf {u}\\&\ge (1 - \varepsilon _2)(\widehat{\mathbf {Q}}_{A_1} - \widehat{\mathbf {Q}}_{A_2}) + \delta \varepsilon _2 \mathbf {u}\\&\ge -(1 - \varepsilon _2)\Vert \widehat{\mathbf {Q}}_{A_2} - \widehat{\mathbf {Q}}_{A_1}\Vert \#(E)\mathbf {u}+ \delta \varepsilon _2\mathbf {u}\\&\ge -\#(E)\varepsilon _3\mathbf {u}+ \delta \varepsilon _2\mathbf {u}= \mathbf {0}.&\text {(by } (5.6) \end{aligned}$$

Let $\lambda \in [(1 + \delta ) \lambda _1,(1 + 2 \delta ) \lambda _1]$ be a rational number, so that $\lambda \mathbf {S}_{A_1} \ge \mathbf {S}_{A_2}$. We let $\mathbf {S}_{\lambda A_1} = \lambda \mathbf {S}_{A_1}$, and we define $B\mapsto \mathbf {S}_B$ on the interval $[A_2,\lambda A_1]$ by linear interpolation:

$$\begin{aligned} \mathbf {S}_B = \mathbf {S}_{A_2} + \frac{B - A_2}{\lambda A_1 - A_2}(\lambda \mathbf {S}_{A_1} - \mathbf {S}_{A_2}) \quad \text { for all }B\in [A_2,\lambda A_1], \end{aligned}$$

and as before we let $\widehat{\mathbf {S}}_B = B^{-1} \mathbf {S}_B$. Then $\widehat{\mathbf {S}}_{\lambda A_1} = \widehat{\mathbf {S}}_{A_1}$, so there is a unique exponentially $\lambda$-periodic extension $\widehat{\mathbf {S}}:(0,\infty )\rightarrow \mathcal P$. We let $\mathbf {S}_B = B \widehat{\mathbf {S}}_B$, and note that $\mathbf {S}$ is increasing.

Fix $B\in [\lambda A_1, C\lambda A_1]$. Since $\lambda _1 \ge C$, we havewhere $X \sim _+Y$ means that the distance between *X* and *Y* tends to zero as the appropriate limit is taken. Similar logic applies if $B\in [C^{-1} A_1,A_1]$, and the cases $B\in [A_1,A_2]$ and $B\in [A_2,\lambda A_1]$ are even easier. So

5.7 $$\begin{aligned} \sup _{B\in [C^{-1} A_1,C\lambda A_1]} \Vert \widehat{\mathbf {S}}_B - \widehat{\mathbf {Q}}_B\Vert \xrightarrow [\varepsilon _2,\delta \rightarrow 0]{} 0. \end{aligned}$$

For each *N*, let $k = k_N \in \mathbb {N}$ be chosen so that $\lambda ^{-k} N \in [1,\lambda ]$. After extracting a subsequence from the sequence along which (5.5) converges, we can assume that

5.8 $$\begin{aligned} \lambda ^{-k} N \dashrightarrow x \in [1,\lambda ]. \end{aligned}$$

Now let $\psi :\mathbb {R}\rightarrow {[0,\infty )}$ be a smooth approximation of the Dirac delta function, let

$$\begin{aligned} \widehat{\mathbf {T}}_{x B} = \int \widehat{\mathbf {S}}_{e^t B} \psi (t)\;\mathrm {d}t, \end{aligned}$$

and let $\mathbf {t}_b = (\partial /\partial b)[b\widehat{\mathbf {T}}_b]$. Then $\mathbf {t}\in \mathcal R_\lambda$, and by choosing $\psi$ appropriately we can guarantee

5.9 $$\begin{aligned} \sup _{B > 0} \Vert \widehat{\mathbf {T}}_{x B} - \widehat{\mathbf {S}}_B\Vert < \varepsilon _2. \end{aligned}$$

Finally, let $\mathbf {r}\in \mathcal Q_\lambda$ be an approximation of $\mathbf {t}$, such that $\mathbf {r}_b \in \mathcal P^*$ for all $b > 0$, and

5.10 $$\begin{aligned} \sup _{b > 0} \Vert \mathbf {r}_b - \mathbf {t}_b\Vert < \varepsilon _2. \end{aligned}$$

Now fix $B\in [1,\lambda ]$, let *N* be large, and let $k = k_N$. Let $k' \in \mathbb {Z}$ be chosen so that $N A_1 \le \lambda ^{k'} B \le N \lambda A_1$. Now fix $M \in [C^{-1} \lambda ^{k'} B, C \lambda ^{k'} B]$, and let $B' = M/N$. By our choice of $k'$, we have $C^{-1} A_1 \le B' \le C\lambda A_1$. Thuswhich completes the proof of the claim. $\square$

Now fix $\lambda > 1$, $B\in [1,\lambda ]$, and $k\in \mathbb {N}$. For each $i\in D$, let $N_i = \lfloor \lambda ^k B_i\rfloor$, where $B_i$ is given by (2.10). Since $\chi _i$ is bounded from above and below on $\mathcal P$, there exists a constant $C \ge 1$ (independent of $\lambda$, *B*, and *k*) such that $N_i \in [C^{-1} \lambda ^k B,C \lambda ^k B]$. Fix $\varepsilon > 0$ and let $1 < \lambda \in \mathbb {Q}$ and $\mathbf {r}\in \mathcal Q_\lambda$ be as in Claim 5.1. Then

$$\begin{aligned} X_i(\omega \upharpoonleft {N_i}) \;&=_{\phantom {\times }}-\sum _{n = 1}^{N_i} \log |\phi _{\omega _n,i}'| = \chi _i(\mathbf {P}_{N_i})\\&\sim _\times \chi _i(\mathbf {R}_{N_i})&\text {(as } \varepsilon \rightarrow 0\text {)}\\&\sim _\times \chi _i(\mathbf {R}_{\lambda ^k B_i})&\text {(as } k\rightarrow \infty \text {)}\\&=_{\phantom {\times }}\lambda ^k \chi _i(\mathbf {R}_{B_i}) = \lambda ^k B,&\text {(by } (2.10)\text {)} \end{aligned}$$

where $X \sim _\times Y$ means that $X/Y \rightarrow 1$ as the appropriate limit is taken. So for some $\delta _2 > 0$ such that $\delta _2 \rightarrow 0$ as $\varepsilon \rightarrow 0$ and $k \rightarrow \infty$, we have

$$\begin{aligned} X_i(\omega \upharpoonleft {N_i}) \ge (1 - \delta _2)\lambda ^k B. \end{aligned}$$

Letting $\rho _k = \exp (-(1 - \delta _2) \lambda ^k B)$, we have $B_\omega (N_1,\ldots ,N_d) \subset \pi ^{-1}(B(\pi (\omega ),\rho _k))$ (cf. (4.2)) and thus

5.11 $$\begin{aligned} -\log \nu _\mathbf {r}\big (B(\pi (\omega ),\rho _k)\big )&\le -\log \mu _\mathbf {r}\big (B_\omega (N_1,\ldots ,N_d)\big )\nonumber \\&= -\sum _{n\in \mathbb {N}} \log \mathbf {r}_n([\omega _n]_{\{i\in D : n\le N_i\}}). \end{aligned}$$

In order to estimate the right-hand side, let $\mathbf {s}:(0,\infty )\rightarrow \mathcal P$ be a piecewise constant and exponentially periodic approximation of $\mathbf {r}$. Let *F* denote the range of $\mathbf {s}$, and note that *F* is finite. Then since $\mathbf {r}_b \in \mathcal P^*$ for all $b > 0$, we can continue the calculation as follows:

$$\begin{aligned}&\sim _\times -\sum _{n\in \mathbb {N}} \log \mathbf {s}_n([\omega _n]_{\{i\in D : n\le N_i\}})&\text {(as } \mathbf {s}\rightarrow \mathbf {r}\text {)}\\&=_{\phantom {\times }}-\sum _{{\emptyset }\ne I \subset D} \sum _{\mathbf {t}\in F} \sum _{\begin{array}{c} n\in \mathbb {N}\\ \mathbf {s}_n = \mathbf {t}\\ \{i\in D : n\le N_i\} = I \end{array}} \log \mathbf {t}([\omega _n]_I). \end{aligned}$$

Now for each $\emptyset \ne I \subset D$ and $\mathbf {t}\in F$, the set

$$\begin{aligned} \big \{n \ge \varepsilon \lambda ^k B : \mathbf {s}_n = \mathbf {t}, \{i \in D : n \le N_i\} = I \big \} \end{aligned}$$

can be written as the union of at most $C_2$ disjoint intervals, where $C_2$ depends only on $\varepsilon$ and $\mathbf {s}$. Write this collection of intervals as $\mathcal I(I,\mathbf {t})$.

We continue the calculation begun in (5.11), using the notation $k\dashrightarrow \infty$ to denote convergence along the sequence tending to the liminf in (5.4):

$$\begin{aligned}&\sim _\times -\sum _{{\emptyset }\ne I \subset D} \sum _{\mathbf {t}\in F} \sum _{\begin{array}{c} n\ge \varepsilon \lambda ^k B \\ \mathbf {s}_n = \mathbf {t}\\ \{i\in D : n\le N_i\} = I \end{array}} \log \mathbf {t}([\omega _n]_I)&\text {(as } \varepsilon \rightarrow 0\text {)}\\&=_{\phantom {\times }}-\sum _{{\emptyset }\ne I \subset D} \sum _{\mathbf {t}\in F} \sum _{(M_1,M_2] \in \mathcal I(I,\mathbf {t})} \int \log \mathbf {t}([{\mathbf {a}}]_I) \;\mathrm {d}[\mathbf {P}_{M_2} - \mathbf {P}_{M_1}]({\mathbf {a}})\\&\sim _\times -\sum _{{\emptyset }\ne I \subset D} \sum _{\mathbf {t}\in F} \sum _{(M_1,M_2] \in \mathcal I(I,\mathbf {t})} \int \log \mathbf {t}([{\mathbf {a}}]_I) \;\mathrm {d}[\mathbf {R}_{M_2} - \mathbf {R}_{M_1}]({\mathbf {a}})&\text {(as } \varepsilon \rightarrow 0 \text { and } k\dashrightarrow \infty \text {)}\\&=_{\phantom {\times }}-\sum _{n \ge \varepsilon \lambda ^k B} \int _n^{n + 1} \int \log \mathbf {s}_n([{\mathbf {a}}]_{\{i\in D : n \le N_i\}}) \;\mathrm {d}\mathbf {r}_b({\mathbf {a}}) \;\mathrm {d}b\\&\sim _\times -\sum _{n\in \mathbb {N}} \int _n^{n + 1} \int \log \mathbf {r}_n([{\mathbf {a}}]_{\{i\in D : n \le N_i\}}) \;\mathrm {d}\mathbf {r}_b({\mathbf {a}}) \;\mathrm {d}b&\text {(as } \varepsilon \rightarrow 0 \text { and } \mathbf {s}\rightarrow \mathbf {r}\text {)}\\&\sim _\times -\iint \log \mathbf {r}_b([{\mathbf {a}}]_{\{i\in D : b \le \lambda ^k B_i\}}) \;\mathrm {d}\mathbf {r}_b({\mathbf {a}}) \;\mathrm {d}b&\text {(as } k\rightarrow \infty \text {)}\\&=_{\phantom {\times }}\int h(\{i \in D : b \le \lambda ^k B_i\};\mathbf {r}_b) \;\mathrm {d}b = \lambda ^k B \delta (\mathbf {r},B). \end{aligned}$$

Dividing by the asymptotic $\lambda ^k B \sim _\times -\log (\rho _k)$ (valid as $\varepsilon \rightarrow 0$) and letting $k\dashrightarrow \infty$ and $\mathbf {s}\rightarrow \mathbf {r}$ shows that

$$\begin{aligned} \underline{\mathrm{d}}(\pi (\omega ),\nu _\mathbf {r}) \le \liminf _{k\rightarrow \infty } \frac{\log \nu _\mathbf {r}\big (B(\pi (\omega ),\rho _k)\big )}{\log (\rho _k)} \le (1 + o(1)) \delta (\mathbf {r},B), \end{aligned}$$

where the *o*(1) term decays to zero as $\varepsilon \rightarrow 0$. Taking the infimum over $B\in [1,\lambda ]$ gives

$$\begin{aligned} \underline{\mathrm{d}}(\pi (\omega ),\nu _\mathbf {r}) \le (1 + o(1)) \delta (\mathbf {r}), \end{aligned}$$

which proves that $\pi (\omega ) \in S_1$, demonstrating (5.1).

Now we prove (5.2). Let $\Omega$ be the set of all $\omega \in E^\mathbb {N}$ such that the limit $\lim _{N\rightarrow \infty } {\widehat{\mathbf {P}}}_N$ exists, where ${\widehat{\mathbf {P}}}_N \in \mathcal P$ is given by (5.3). By the ergodic theorem, every invariant measure gives full measure to $\Omega$, so ${\dim _D}(\Phi ) \le {\dim _H}(\pi (\Omega ))$. Now for each $\omega \in \Omega$, we can choose $\mathbf {r}= \mathbf {p}\in \mathcal Q_1\cap \mathcal P^*$ satisfying (5.4), namely any approximation to the limit $\lim _{N\rightarrow \infty } {\widehat{\mathbf {P}}}_N$. The remainder of the argument (i.e. everything after the proof of Claim 5.1) is still applicable, and shows that $\underline{\mathrm{d}}(\pi (\omega ),\nu _\mathbf {p}) \le (1 + o(1)) \delta (\mathbf {p})$, so $\pi (\omega ) \in S_2$. Since $\omega$ was arbitrary, we have $\pi (\Omega ) \subset S_2$, demonstrating (5.2). $\square$

## Continuity of dimension functions

In this section we prove the continuity of the Hausdorff and dynamical dimensions as functions of the defining IFS, i.e. Theorem 2.9.

### Theorem 6.1

(Generalization of Theorem 2.9) The functions

$$\begin{aligned} \Phi&\mapsto \sup _{\mathbf {r}\in \mathcal R} \delta (\mathbf {r}),&\Phi&\mapsto \sup _{\mathbf {p}\in \mathcal P} \delta (\mathbf {p}) \end{aligned}$$

are continuous on the space of all diagonal IFSes.

### Proof

It is easy to see that the maps

$$\begin{aligned} (\Phi ,i,\mathbf {p})&\mapsto \chi _i(\mathbf {p}),&(\Phi ,I,\mathbf {p})&\mapsto h_I(\mathbf {p}) \end{aligned}$$

are continuous. Applying (2.11) shows that the map

$$\begin{aligned} (\Phi ,\mathbf {p}) \mapsto \delta (\mathbf {p}) \end{aligned}$$

is continuous. Since $\mathcal P$ is compact, it follows that the map $\Phi \mapsto \sup _{\mathbf {p}\in \mathcal P} \delta (\mathbf {p})$ is continuous.

Now if we endow $\mathcal R$ with the topology of locally uniform convergence, then the maps

$$\begin{aligned} (\Phi ,i,\mathbf {r},B)&\mapsto B_i,&(\Phi ,\mathbf {r},B)&\mapsto \delta (\mathbf {r},B) \end{aligned}$$

are continuous. Since the infimum in (2.8) is taken over a compact set, it follows that the map

$$\begin{aligned} (\Phi ,\lambda ,\mathbf {r}) \mapsto \delta (\mathbf {r}) \end{aligned}$$

is continuous. Here we need to include $\lambda$ as an input because of its appearance in the formula (2.8).

Now we define the *exponential Lipschitz constant* of a cycle $\mathbf {r}\in \mathcal R$ to be the Lipschitz constant of the periodic function $t\mapsto \mathbf {r}_{\exp (t)}$. Note that although some elements of $\mathcal R$ have infinite exponential Lipschitz constant, we can choose the countable dense subsets $\mathcal Q_\lambda \subset \mathcal R_\lambda$ appearing in the proof of Theorem 3.3 so that all elements of $\mathcal Q{\, \mathop {=}\limits ^{\mathrm {def}}\, }\bigcup _{1 \le \lambda \in \mathbb {Q}} \mathcal Q_\lambda$ have finite exponential Lipschitz constant. For each $k > 1$, let $\mathcal R_{\lambda ,k}$ (resp. $\mathcal Q_{\lambda ,k}$) denote the set of all cycles $\mathbf {r}\in \mathcal R_\lambda$ (resp. $\mathbf {r}\in \mathcal Q_\lambda$) with exponential Lipschitz constant $\le k$. Then by the Arzela–Ascoli theorem, the set

$$\begin{aligned} \coprod _{\lambda \in [1,k]} \mathcal R_{\lambda ,k} = \{(\lambda ,\mathbf {r}) : \lambda \in [1,k], \; \mathbf {r}\in \mathcal R_{\lambda ,k}\} \end{aligned}$$

is compact, and thus for each *k* the map

$$\begin{aligned} \Phi \mapsto \delta _k {\, \mathop {=}\limits ^{\mathrm {def}}\, }\sup _{\mathbf {r}\in \bigcup _{\lambda \in [1,k]} \mathcal R_{\lambda ,k}} \delta (\mathbf {r}) \end{aligned}$$

is continuous. To complete the proof, we need to show that the convergence

$$\begin{aligned} \delta _k \xrightarrow [k\rightarrow \infty ]{} \sup _{\mathbf {r}\in \mathcal R} \delta (\mathbf {r}) \end{aligned}$$

is locally uniform with respect to $\Phi$.

Indeed, fix $\varepsilon > 0$, and let $0< \varepsilon _3< \varepsilon _2 < 1$ be as in the proof of Claim 5.1. Then:

- The numbers $T_1,T_2 \in \mathbb {R}$ appearing in the proof of Claim 5.1 may be chosen so that $\rho _1 {\, \mathop {=}\limits ^{\mathrm {def}}\, }T_2 - T_1$ is bounded depending only on *C*, $\varepsilon _3$, and $\#(E)$. Since $\lambda$ can be bounded in terms of $\rho _1$, this shows that the $\lambda$ appearing in the conclusion of Claim 5.1 can be bounded in terms of the *C* and $\varepsilon$ that appear in the hypotheses. Now *C* depends only on the maximum and minimum of the function $D\times \mathcal P\ni (i,\mathbf {p}) \mapsto \chi _i(\mathbf {p})$, so it is bounded when $\Phi$ ranges over a compact set. So $\lambda$ can be bounded in terms of $\varepsilon$, assuming that $\Phi$ ranges over a compact set.
- The exponential Lipschitz constant of the function $\mathbf {t}$ appearing in the proof of Claim 5.1 can be bounded in terms of the $C^2$ norm of the smooth function $\psi$. The function $\psi$ depends only on $\varepsilon _2$, which in turn depends only on $\varepsilon$. Moreover, an approximation $\mathbf {r}\in \mathcal Q_\lambda$ of $\mathbf {t}$ satisfying (5.10) can be found with exponential Lipschitz constant bounded in terms of the Lipschitz norm of $\mathbf {t}$. So the exponential Lipschitz constant of $\mathbf {r}$ is bounded in terms of $\varepsilon$.
- The rate of convergence of the *o*(1) term to 0 at the end of the proof of Theorem 3.3 is locally uniform with respect to $\Phi$ as $\varepsilon \rightarrow 0$.

Thus the proof of Theorem 3.3 actually shows that

$$\begin{aligned} {\dim _H}(\Phi ) \le {\dim _H}\left( \bigcap _{\varepsilon> 0} \bigcup _{\lambda \in \mathbb {Q}\cap [1,k(\varepsilon )]} \bigcup _{\mathbf {r}\in \mathcal Q_{\lambda ,k(\varepsilon )}} S_{\mathbf {r},\varepsilon }\right) \le \inf _{\varepsilon > 0} [\delta _{k(\varepsilon )} + \varepsilon ] \end{aligned}$$

for some function *k* that can be taken to be independent of $\Phi$ as $\Phi$ ranges over a compact set. Thus if $\Lambda _\Phi$ is good, then

6.1 $$\begin{aligned} \delta _{k(\varepsilon )} \ge \sup _{\mathbf {r}\in \mathcal R} \delta (\mathbf {r}) - \varepsilon , \end{aligned}$$

which completes the proof in this case. If $\Lambda _\Phi$ or its perturbations are not good, then we may justify the inequality (6.1) by appealing to the existence of a good sponge $\Lambda _\Psi$ with good perturbations, indexed by the same set *E*, such that $|\psi _{i,a}'| = |\phi _{i,a}'|^\alpha$ for all $i\in D$ and $a\in A_i$. Here $\alpha > 0$ must be chosen large enough so that $\sum _{a\in A_i} |\phi _{i,a}'|^\alpha < 1$ for all $i\in D$, which guarantees the existence of a base IFS $\Psi _i$ whose perturbations satisfy the open set condition. It is readily verified that $\delta _\Psi (\mathbf {r}) = \delta _\Phi (\mathbf {r})/\alpha$ for all $\mathbf {r}\in \mathcal R$, so that (6.1) holds for $\Phi$ if and only if it holds for $\Psi$. $\square$

## Special cases where ${\dim _H}(\Phi ) = {\dim _D}(\Phi )$

In this section we give new proofs of Theorems 2.4 and 2.5, i.e. equality of the Hausdorff and dynamical dimensions in certain special cases, based on the results of the previous sections. Both of the theorems can now be stated in somewhat greater generality than they were in the introduction.

### Theorem 7.1

(Generalization of Theorem 2.5) Let $\Lambda _\Phi$ be a good sponge such that for all $i\in D$, the map $A_i \ni a \mapsto |\phi _{i,a}'|$ is constant. Then ${\dim _H}(\Phi ) = {\dim _D}(\Phi )$.

### Proof

Fix $\mathbf {r}\in \mathcal R$, and we will show that $\delta (\mathbf {r}) \le {\dim _D}(\Phi )$. For each $i\in D$, let $r_i > 0$ be the constant such that $|\phi _{i,a}'| = r_i$ for all $a\in A_i$, and let $X_i = -\log (r_i)$. For all $B > 0$ and $i\in D$, we have

$$\begin{aligned} B = \chi _i(\mathbf {R}_{B_i}) = X_i B_i, \end{aligned}$$

i.e. $B_i = B/X_i$. Now without loss of generality suppose that $X_1 \le \cdots \le X_d$. Then

$$\begin{aligned} \delta (\mathbf {r})&\le \frac{1}{\log (\lambda )} \int _1^\lambda \delta (\mathbf {r},B) \;\frac{\mathrm {d}B}{B}&\text {(by }(2.8)\text {)}\\&\le \frac{1}{\log (\lambda )} \int _1^\lambda \sum _{i\in D} \frac{h(I_{\le i}\upharpoonleft I_{\le i - 1};{\widehat{\mathbf {R}}}_{B_i})}{\chi _i({\widehat{\mathbf {R}}}_{B_i})} \;\frac{\mathrm {d}B}{B}&\text {(by }(2.12)\text {)}\\&= \frac{1}{\log (\lambda )} \int _1^\lambda \sum _{i\in D} \frac{h(I_{\le i}\upharpoonleft I_{\le i - 1};{\widehat{\mathbf {R}}}_A)}{\chi _i({\widehat{\mathbf {R}}}_A)} \;\frac{\mathrm {d}A}{A}&\text {(letting } A = B_i\text {)}\\&= \frac{1}{\log (\lambda )} \int _1^\lambda \delta ({\widehat{\mathbf {R}}}_A) \;\frac{\mathrm {d}A}{A}&\text {(by }(2.13)\text {)}\\&\le \frac{1}{\log (\lambda )} \int _1^\lambda {\dim _D}(\Phi ) \;\frac{\mathrm {d}A}{A} = {\dim _D}(\Phi ).&\text {(by }(2.2) \hbox { and } (2.11)\text {)} \end{aligned}$$

The key step in this proof is the substitution $A = B_i = B/X_i$, which is valid because $\mathrm {d}B_i/B_i = \mathrm {d}B/B$. In general, when $B_i$ and *B* are only related by the formula (2.10), the relation $\mathrm {d}B_i/B_i = \mathrm {d}B/B$ is not valid, and that is the reason that this proof does not work in the general case. $\square$

### Theorem 7.2

(Generalization of Theorem 2.4) For every good sponge $\Lambda _\Phi \subset [0,1]^d$, we have

$$\begin{aligned} {\dim _H}(\Phi ) \le \max (1,d - 1) {\dim _D}(\Phi ). \end{aligned}$$

In particular, if $d \le 2$ then ${\dim _H}(\Phi ) = {\dim _D}(\Phi )$.

### Proof

Fix $\mathbf {r}\in \mathcal R$, and we will show that $\delta (\mathbf {r}) \le \max (1,d - 1){\dim _D}(\Phi )$. For each $B > 0$ and $i\in D$ we let

$$\begin{aligned} J_{i,B} = \{j\in D : \chi _j({\widehat{\mathbf {R}}}_B) \le ^* \chi _i({\widehat{\mathbf {R}}}_B)\}, \end{aligned}$$

where the star on the inequality means that in the case of a tie, we determine whether or not the inequality is true using an arbitrary but fixed “tiebreaker” total order $\prec$ on *D*: we declare the inequality to be true if $j \prec i$, and false if $j \succ i$. Then we let

$$\begin{aligned} f_i(B) = \frac{h(J_{i,B}\cup \{i\} \upharpoonleft J_{i,B};{\widehat{\mathbf {R}}}_B)}{\chi _i({\widehat{\mathbf {R}}}_B)}\cdot \end{aligned}$$

Now,

- If $\chi _1({\widehat{\mathbf {R}}}_B) \le ^* \cdots \le ^* \chi _d({\widehat{\mathbf {R}}}_B)$, then $J_{i,B} = I_{\le i - 1}$, and so by Theorem 2.7 and Proposition 2.16,
  7.1 $$\begin{aligned} {\dim _D}(\Phi ) \ge \delta ({\widehat{\mathbf {R}}}_B) = \sum _{i\in D} f_i(B). \end{aligned}$$
- If $B_1 \ge ^* \cdots \ge ^* B_d$, then $\chi _j(\mathbf {R}_{B_i}) \le ^* \chi _i(\mathbf {R}_{B_i}) \le ^* \chi _{j'}(\mathbf {R}_{B_i})$ for all $j< i < j'$, so $J_{i,B_i} = I_{\le i - 1}$, and thus by Proposition 2.16, we have
  7.2 $$\begin{aligned} \delta (\mathbf {r}) \le \delta (\mathbf {r},B) \le \sum _{i\in D} f_i(B_i), \end{aligned}$$
  where $B_1,\ldots ,B_d > 0$ are as in (2.10).

Both of these hypotheses can be attained by appropriately permuting *D*, assuming that the tiebreaker total order is getting permuted as well. So since the formulas (7.1) and (7.2) are invariant under permutations of *D*, they are true regardless of how the numbers $\chi _i({\widehat{\mathbf {R}}}_B)$ ($i\in D$) and $B_i$ ($i\in D$) are ordered.

Now fix $\varepsilon > 0$, and let $B > 0$ be chosen so that $f_1(B_1) \le \inf (f_1) + \varepsilon$. (This is possible because the map $B \mapsto B_1$ is a homeomorphism of $(0,\infty )$.) If $d \ge 2$, then we get

$$\begin{aligned} f_1(B_1) + f_2(B_2) \le f_1(B_2) + \varepsilon + f_2(B_2) \le {\dim _D}(\Phi ) + \varepsilon \end{aligned}$$

and thus

$$\begin{aligned} \delta (\mathbf {r}) \le \sum _{i\in D} f_i(B_i) \le {\dim _D}(\Phi ) + \varepsilon + \sum _{i = 3}^d f_i(B_i) \le (d - 1) {\dim _D}(\Phi ) + \varepsilon . \end{aligned}$$

Since $\mathbf {r}$ and $\varepsilon$ were arbitrary, we get ${\dim _H}(\Phi ) \le (d - 1){\dim _D}(\Phi )$. If $d = 1$, then ${\dim _H}(\Phi ) = {\dim _D}(\Phi )$, so in any case ${\dim _H}(\Phi ) \le \max (1,d - 1){\dim _D}(\Phi )$. $\square$

### Remark 7.3

This new way of proving Theorem 2.4 sheds light on the question of why there is a difference between the two-dimensional and three-dimensional settings. Namely, since we used the assumption $d = 2$ only at the last possible moment, the proof clarifies exactly how the assumption is needed in the argument.

At a very abstract level, the difference between the two-dimensional and three-dimensional case can be described as follows: The Hausdorff dimension of a “homogeneous” non-invariant measure (such as a pseudo-Bernoulli measure) is equal to the lim inf of its dimension at different length scales. At each length scale, the dimension is equal to the sum of the coordinatewise dimensions at that scale. So if $\delta _i$ is the coordinatewise dimension as a function of the length scale $\rho$, then

$$\begin{aligned} {\dim _H}(\text {non-invariant measure}) = \liminf _{\rho \rightarrow 0} \sum _i \delta _i(\rho ). \end{aligned}$$

Now, the existence of this non-invariant homogeneous measure will allow us to deduce the existence of certain invariant measures, namely there exist continuously varying tuples of length scales $(\rho _1,\ldots ,\rho _d)\rightarrow 0$ such that there is some invariant measure which for all *i* has the same behavior as the non-invariant measure in coordinate *i* and length scale $\rho _i$. The dimension of such a measure would be

$$\begin{aligned} {\dim _H}(\text {invariant measure}) = \sum _i \delta _i(\rho _i). \end{aligned}$$

Obviously, the problem with comparing these two formulas is that the $\rho _i$s may be different from each other. In dimension 1, there is only one number $\rho _i$ so there is no issue. But we can handle one more dimension using the fact that the first formula has a lim inf instead of a lim sup. Namely, we can choose a value of $\rho$ so as to minimize one of the numbers $\delta _i(\rho )$, for concreteness say $\delta _1(\rho )$. This handles the first coordinate, and we can handle the second coordinate by choosing the pair $(\rho _1,\rho _2)$ so that $\rho _2 = \rho$. But there is no way to handle any more coordinates.

One aspect of this explanation is that it implies that the reason we can handle two coordinates instead of just one is that we are considering the Hausdorff dimension, which corresponds to a lim inf, rather than the packing dimension, which corresponds to a lim sup. It is well-known that the Hausdorff and packing dimensions of a self-affine set can be different even in two dimensions; see e.g. [28, Theorem 4.6] together with [38, Proposition 2.2(i)]. This is in contrast to the situation for finite conformal IFSes, where the Hausdorff and packing dimensions are always the same [31, Lemma 3.14].

## Construction of dimension gap sponges

In this section we prove the main result of this paper, the existence of sponges with a dimension gap, viz. Theorem 2.8. Before starting the proof, we give a sketch to convey the main ideas. In the sketch we write down formulas without giving any justification, since these formulas will be justified in detail in the real proof.

### CONVENTION 1

We denote the product of two matrices $\mathbf {A}$ and $\mathbf {B}$ by $\mathbf {A}\cdot \mathbf {B}$. It should not be confused with the scalar product of two vectors $\mathbf {v}$ and $\mathbf {w}$, which we denote by $\langle \mathbf {v},\mathbf {w}\rangle$.

**Fig. 2:**
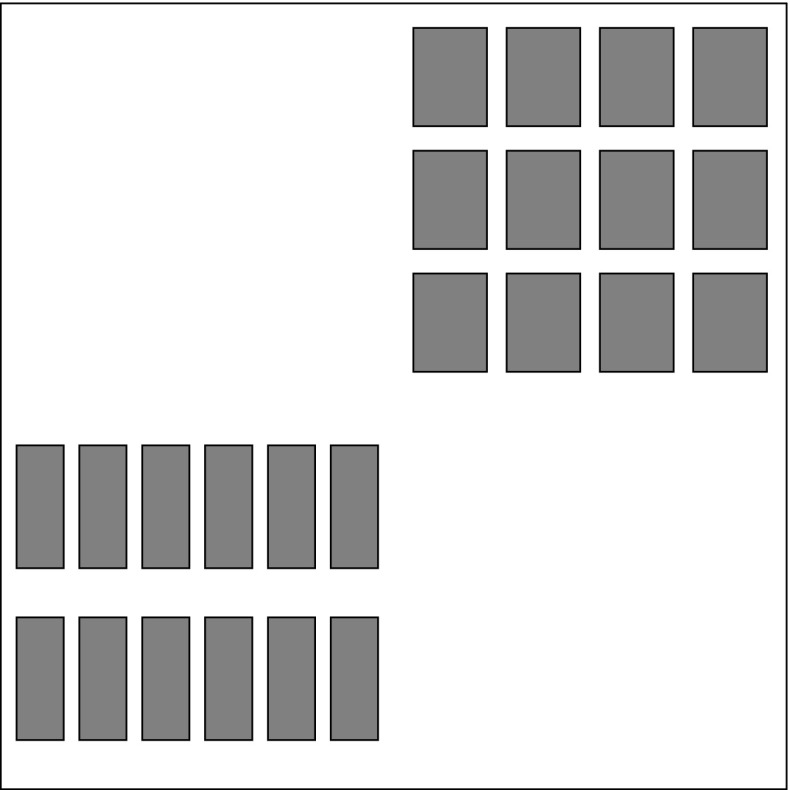
An example of the disjoint-union-of-product-IFSes construction, with $\#(D) = \#(J) = 2$. In the actual proof of Theorem 2.8 we have $\#(D) = \#(J) =3$

### Proof Sketch of Theorem 2.8

The goal is to find a diagonal IFS $\Phi = (\phi _a)_{a\in E}$ on $[0,1]^3$ and a cycle $\mathbf {r}\in \mathcal R$ such that ${\dim _H}(\nu _\mathbf {r}) > {\dim _D}(\Phi )$. The IFS will be of a special form: it will be the disjoint union of three sub-IFSes, each of which will be the direct product of three similarity IFSes on [0, 1] (cf. Fig. 2). Letting $D = J = \{1,2,3\}$, we can write $\Phi = \coprod _{j\in J} \prod _{i\in D} \Phi _{i,j}$, where for each $i\in D$ and $j\in J$, $\Phi _{i,j} = (\phi _{i,j,a})_{a\in E_{i,j}}$ is a similarity IFS on [0, 1] consisting of similarities all with the same contraction ratio. The properties of the overall IFS $\Phi$ are determined up to some fudge factors by the entropy and Lyapunov exponents of the component IFSes $\Phi _{i,j}$ ($i\in D$, $j\in J$), which we denote by $H_{i,j}$ and $X_{i,j}$, respectively. (In the actual proof, the entropy and Lyapunov exponent of $\Phi _{i,j}$ will only be approximately proportional to $H_{i,j}$ and $X_{i,j}$, rather than equal.) The matrices $\mathbf {H}= (H_{i,j})$ and $\mathbf {X}= (X_{i,j})$ can be more or less arbitrary, subject to the restriction that $0< H_{i,j} < X_{i,j}$, which describes the fact that the dimension of the limit set of $\Phi _{i,j}$ must be strictly between 0 and 1. To make the overall IFS satisfy the coordinate ordering condition, the further restriction $X_{i,j} < X_{i + 1,j}$ is also needed.

Once the relation between $\Phi$ and the matrices $\mathbf {H}$ and $\mathbf {X}$ has been established, ${\dim _D}(\Phi )$ can be estimated based on $\mathbf {H}$ and $\mathbf {X}$. The maximum of the function $\mathbf {p}\mapsto \delta (\mathbf {p})$ is always attained at points of the form $\sum _{j\in J} q_j \mathbf {u}_j$, where $\mathbf {u}_j$ denotes the normalized uniform measure on $E_j {\, \mathop {=}\limits ^{\mathrm {def}}\, }\prod _{i\in D} E_{i,j}$, i.e. $\mathbf {u}_j = \#(E_j)^{-1} \sum _{{\mathbf {a}}\in E_j} \delta _{\mathbf {a}}$, and $\mathbf {q}= (q_1,q_2,q_3) \in \mathbb {R}^J$ is a probability vector. Equivalently, the maximum is attained at $\mathbf {M}\cdot \mathbf {q}$ for some $\mathbf {q}\in \Delta$, where $\Delta \subset \mathbb {R}^J$ is the space of probability vectors on *J* and $\mathbf {M}\cdot \mathbf {e}_j = \mathbf {u}_j$ for all $j\in J$. Here and hereafter $(\mathbf {e}_j)_{j\in J}$ denotes the standard basis of $\mathbb {R}^J$. To make things simpler later, we will choose $\mathbf {H}$ and $\mathbf {X}$ so that we can be even more precise: the maximum of $\mathbf {p}\mapsto \delta (\mathbf {p})$ is attained at $\mathbf {p}= \mathbf {M}\cdot \mathbf {u}$, where $\mathbf {u}= (1/3,1/3,1/3)\in \Delta$ is the normalized uniform measure on *J*.

**Fig. 3:**
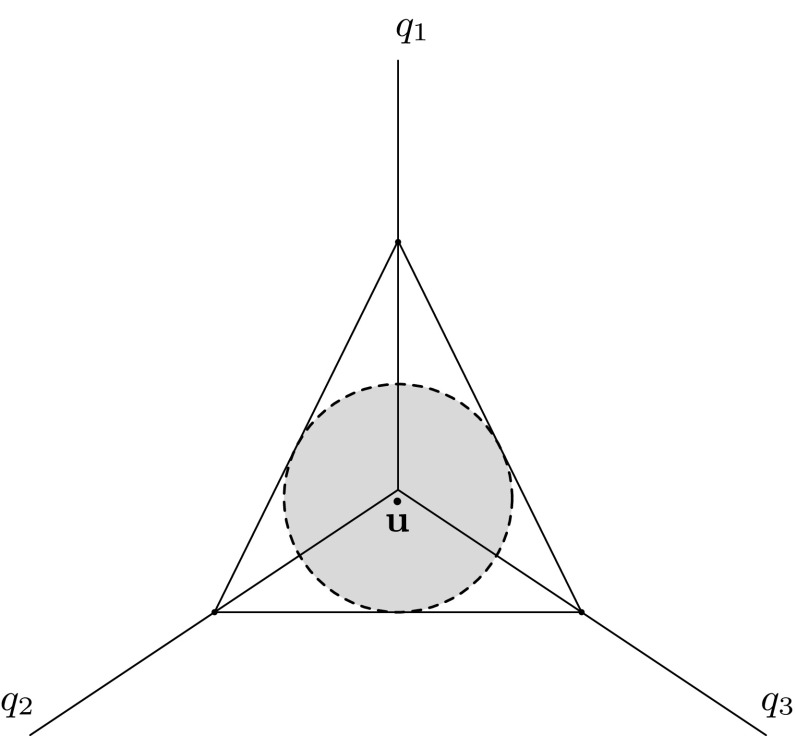
The inscribed circle of the simplex $\Delta$, which represents the trajectory of $\mathbf {s}$. This trajectory geometrically represents the non-invariant/pseudo-Bernoulli measure that we prove has dimension strictly greater than the dynamical dimension, while its center $\mathbf {u}$ represents the invariant/Bernoulli measure of maximal dimension

Next, let us describe the cycle $\mathbf {r}\in \mathcal R$ for which we will prove that ${\dim _H}(\nu _\mathbf {r}) > {\dim _D}(\Phi )$. Its range will consist of probability vectors of the form $\mathbf {M}\cdot \mathbf {q}$ with $\mathbf {q}\in \Delta$, i.e. those probability vectors which were considered candidates for the maximum of $\mathbf {p}\mapsto \delta (\mathbf {p})$ in the previous paragraph. So we can write $\mathbf {r}_b = \mathbf {M}\cdot \mathbf {s}_b$, where $\mathbf {s}:(0,\infty )\rightarrow \Delta$ is exponentially periodic. The trajectory of $\mathbf {s}$ will be the inscribed circle of the triangle $\Delta$ (cf. Fig. 3), and the exponential period of $\mathbf {s}$ will be $e^{2\pi \gamma }$ for some small number $\gamma > 0$. Formally, we will write

$$\begin{aligned} \mathbf {s}_{\exp (\gamma t)} = \mathbf {z}(t) \end{aligned}$$

where $\mathbf {z}:\mathbb {R}\rightarrow \Delta$ is a unit speed (with respect to angle) parameterization of the inscribed circle of $\Delta$. (In the actual proof, for greater generality we will let $\rho$ denote the period of $\mathbf {z}$, so that in our case $\rho = 2\pi$.)

### Remark 8.1

The fact that the trajectory of $\mathbf {s}$ is a circle is motivated by the fact that $\mathbf {s}$ should be (exponentially) periodic and smooth, and that the “center” of its trajectory should be the maximum of $\mathbf {p}\mapsto \delta (\mathbf {p})$. The fact that the exponential period is close to 1 is motivated by the fact that the “advantage” that non-constant cycles $\mathbf {r}\in \mathcal R$ have over constant points $\mathbf {p}\in \mathcal P$ is the fact that they are “moving”, so to maximize this advantage, it makes sense to maximize the speed of motion. However, the tradeoff is that the dimension gap ${\dim _H}(\nu _\mathbf {r}) - {\dim _D}(\Phi )$ ends up depending proportionally on $\gamma$ as $\gamma \rightarrow 0$ (see (8.1) below), so the size of the dimension gap tends to zero as $\gamma \rightarrow 0$. This is one of the reasons that it is difficult for us to get good lower bounds on the size of the dimension gap; cf. Questions 9.2.

With this setup, after making the additional simplification that $\mathbf {H}_i\cdot \mathbf {u}= 2^{i - 1}$ and $\mathbf {X}_i\cdot \mathbf {u}= 2^i$ for all $i\in D$, where $\mathbf {H}_i$ and $\mathbf {X}_i$ denote the *i*th rows of $\mathbf {H}$ and $\mathbf {X}$, respectively, one finds that the size of the dimension gap is

8.1 $$\begin{aligned} {\dim _H}(\nu _\mathbf {r}) - {\dim _D}(\Phi ) = \gamma \inf _{t\in [0,2\pi ]}\sum _{i\in D} \mathbf {K}_i\cdot \mathbf {Z}(t_{i,0}) + O(\gamma ^2), \end{aligned}$$

where $\mathbf {K}_i = 2^{-i} (\mathbf {H}_i - (1/2)\mathbf {X}_i)$, $\mathbf {Z}:\mathbb {R}\rightarrow \mathbb {R}^J$ is a unit speed parameterization of a certain circle in the plane $P = \{\mathbf {q}\in \mathbb {R}^J : q_1 + q_2 + q_3 = 0\}$, and $t_{i,0}$ is defined by the equation

8.2 $$\begin{aligned} t = t_{i,0} + \mathbf {Y}_i\cdot \mathbf {Z}(t_{i,0}) \;\;\forall i, \end{aligned}$$

where $\mathbf {Y}_i = 2^{-i} \mathbf {X}_i$. So the goal now is to make the coefficient of $\gamma$ in (8.1) positive, while still making sure that the maximum of $\mathbf {p}\mapsto \delta (\mathbf {p})$ is attained at $\mathbf {p}= \mathbf {M}\cdot \mathbf {u}$. This is done most efficiently by assuming that $\mathbf {K}$ and $\mathbf {Y}$ are close to a known value that would lead to the map $\Delta \ni \mathbf {q}\mapsto \delta (\mathbf {M}\cdot \mathbf {q})$ being constant; i.e.

$$\begin{aligned} \mathbf {K}&= \varepsilon {\widehat{\mathbf {K}}},&\mathbf {Y}&= \mathbf {U}+ \varepsilon {\widehat{\mathbf {Y}}}, \end{aligned}$$

where $\mathbf {U}$ is the $3\times 3$ matrix whose entries are all equal to 1, ${\widehat{\mathbf {K}}}$ and ${\widehat{\mathbf {Y}}}$ are matrices chosen so that ${\widehat{\mathbf {K}}}\cdot \mathbf {u}= {\widehat{\mathbf {Y}}}\cdot \mathbf {u}= 0$, and $\varepsilon > 0$ is small. Then the time $t_{i,0}$ defined by (8.2) approaches *t* as $\varepsilon \rightarrow 0$, so the coefficient of $\gamma$ in (8.1) becomes

$$\begin{aligned} \varepsilon ^2\inf _{t\in [0,2\pi ]} {\widehat{\mathbf {K}}}_i\cdot \mathbf {Z}'(t)[-{\widehat{\mathbf {Y}}}_i\cdot \mathbf {Z}(t)] + O(\varepsilon ^3), \end{aligned}$$

so we need the coefficient of $\varepsilon ^2$ in this expression to be positive:

8.3 $$\begin{aligned} \sup _{t\in [0,2\pi ]} ({\widehat{\mathbf {K}}}_i\cdot \mathbf {Z}'(t))({\widehat{\mathbf {Y}}}_i\cdot \mathbf {Z}(t)) < 0. \end{aligned}$$

At the same time, we need the maximum of $\mathbf {p}\mapsto \delta (\mathbf {p})$ to be attained at $\mathbf {p}= \mathbf {M}\cdot \mathbf {u}$; it is enough to check that

8.4 $$\begin{aligned} \sum _{i\in D} {\widehat{\mathbf {K}}}_i&= \mathbf {0},&\sum _{i\in D} ({\widehat{\mathbf {K}}}_i\cdot \mathbf {q})({\widehat{\mathbf {Y}}}_i\cdot \mathbf {q}) > 0 \;\;\forall \mathbf {q}\in \mathbb {R}^J{\setminus }\mathbb {R}\mathbf {u}. \end{aligned}$$

The proof is then completed by finding matrices ${\widehat{\mathbf {K}}}$ and ${\widehat{\mathbf {Y}}}$ that satisfy all these requirements. Intuitively, the difficulty should come in reconciling the requirements (8.3) and (8.4), since the latter is what shows that a constant element of $\Delta$ cannot produce a dimension greater than 3 / 2, while the former is what shows that the nonconstant circular cycle can produce such a dimension gap. However, the requirements are compatible because (8.3) incorporates the geometry of circular motion, in which the derivative $\mathbf {Z}'(t)$ is always orthogonal to $\mathbf {Z}(t)$, while (8.4) cannot incorporate the geometry of any shape because it comes from considering only constant cycles. This completes the proof sketch. $\square$

### Proof of Theorem 2.8

It suffices to consider the case $d = 3$, since a 3-dimensional Barański sponge can be isometrically embedded into any higher dimension. Let $\mathbf {H}= (H_{i,j})$ and $\mathbf {X}= (X_{i,j})$ be $3\times 3$ matrices to be specified later. We think of their rows as being indexed by the set $D {\, \mathop {=}\limits ^{\mathrm {def}}\, }\{1,2,3\}$, while their columns are indexed by $J {\, \mathop {=}\limits ^{\mathrm {def}}\, }\{1,2,3\}$. Here we have made a conceptual distinction between the sets *D* and *J* even though they are set-theoretically the same, because the fact that these two sets have the same cardinality has no relevance until much later in the argument. Geometrically, *D* corresponds to the number of dimensions (i.e. *D* is the set of coordinates), while *J* corresponds to the number of distinct “types” of contractions that we will put into our diagonal IFS. We will assume that

8.5 $$\begin{aligned}&0< H_{i,j}< X_{i,j} \;\;\forall i\in D \;\;\forall j\in J,\nonumber \\&X_{i,j} < X_{i + 1,j} \;\;\forall i = 1,2 \;\;\forall j\in J. \end{aligned}$$

Fix *k* large. For each $i\in D$ and $j\in J$, let $N_{i,j} = \lfloor e^{k H_{i,j}}\rfloor$ and $r_{i,j} = e^{-k X_{i,j}}$, and let $\Phi _{i,j} = (\phi _{i,j,a})_{a\in E_{i,j}}$ be a one-dimensional IFS of contracting similarities satisfying the strong separation condition with respect to [0, 1] such that

(I) $$\phi _{i,j,a}([0,1]) \subset ((j - 1)/3,j/3)$$ for all $$a\in E_{i,j}$$;
(II) $$\#(E_{i,j}) = N_{i,j}$$; and
(III) $|\phi _{i,j,a}'| = r_{i,j}$ for all $a\in E_{i,j}$.

This is possible as long as $N_{i,j} r_{i,j} < 1/3$, which is true for all sufficiently large *k*, since by hypothesis $H_{i,j} < X_{i,j}$.

Now for each $j \in J$, let $E_j = \prod _{i \in D} E_{i,j}$ and $\Phi _j = (\phi _{j,{\mathbf {a}}})_{{\mathbf {a}}\in E_j}$, where $\phi _{j,{\mathbf {a}}}(\mathbf {x}) = (\phi _{i,j,a_i}(x_i))_{i\in D}$. Let

$$\begin{aligned} E = \coprod _{j \in J} E_j = \{(j,{\mathbf {a}}) : j\in J, {\mathbf {a}}\in E_j\}, \end{aligned}$$

and consider the IFS $\Phi = (\phi _{j,{\mathbf {a}}})_{(j,{\mathbf {a}})\in E}$. Note that the second half of condition (8.5) guarantees that $\Phi$ satisfies the coordinate ordering condition with respect to the identity permutation. To emphasize the dependence of $\Phi$ on the parameter *k*, we will sometimes write $\Phi _k$ instead of $\Phi$.

We proceed to estimate ${\dim _D}(\Phi )$ and ${\dim _H}(\Phi )$.

*Estimation of* ${\dim _D}(\Phi )$. For each $i\in D$ and $j \in J$, let ${{\mathrm{Perm}}}(E_{i,j})$ denote the group of permutations of $E_{i,j}$. Then the group $G = \prod _{j \in J} \prod _{i \in D} {{\mathrm{Perm}}}(E_{i,j})$ admits a natural action on *E*, with respect to which the functions $h_I$ ($I\subset D$) and $\chi _i$ ($i\in D$) are invariant. Now let $\mathbf {p}$ be any probability measure on *E*, and let ${\widehat{\mathbf {p}}} = \mu _G*\mathbf {p}$, where $\mu _G$ is the Haar/uniform measure of *G* and $*$ denotes convolution. Note that ${\widehat{\mathbf {p}}}$ is *G*-invariant. Since $h_I$ is superlinear and $\chi _i$ is linear, we have $h_I({\widehat{\mathbf {p}}}) \ge h_I(\mathbf {p})$ and $\chi _i({\widehat{\mathbf {p}}}) = \chi _i(\mathbf {p})$ for all *I* and *i*. Consequently, it follows from (2.11) that $\delta ({\widehat{\mathbf {p}}}) \ge \delta (\mathbf {p})$, so the supremum in (2.2) can be taken over the class of *G*-invariant measures on *E*. Such measures are of the form

$$\begin{aligned} \mathbf {p}= \sum _{j \in J} q_j \mathbf {u}_j, \end{aligned}$$

where $\mathbf {q}= (q_1,q_2,q_3)$ is a probability vector on *J*, and $\mathbf {u}_j$ denotes the normalized uniform measure on $E_j$, i.e. $\mathbf {u}_j = \#(E_j)^{-1} \sum _{{\mathbf {a}}\in E_j} \delta _{\mathbf {a}}$. Equivalently, $\mathbf {p}= \mathbf {M}\cdot \mathbf {q}$, where $\mathbf {M}:\mathbb {R}^J \rightarrow \mathbb {R}^E$ is the linear operator such that $\mathbf {M}\cdot \mathbf {e}_j = \mathbf {u}_j$ for all $j\in J$. Note that for all $I \subset D$, by Lemma 4.2 we have^6^
8.6and for all $$i\in D$$

8.7 $$\begin{aligned} \chi _i(\mathbf {M}\cdot \mathbf {q})&= \sum _{j \in J} q_j \chi _i(\mathbf {u}_j) = \sum _{j \in J} q_j k X_{i,j} = k \mathbf {X}_i\cdot \mathbf {q}. \end{aligned}$$

Here $\mathbf {H}_i$ and $\mathbf {X}_i$ denote the *i*th rows of $\mathbf {H}$ and $\mathbf {X}$, respectively, i.e. $\mathbf {H}_i = \mathbf {e}_i^*\cdot \mathbf {H}$ and $\mathbf {X}_i \mathbf {e}_i^*\cdot \mathbf {X}$, where $(\mathbf {e}_i^*)_{i\in D}$ is the dual of the standard basis of $\mathbb {R}^d$. So by (2.13), we have

8.8 $$\begin{aligned} {\dim _D}(\Phi _k) = \max _{\mathbf {q}\in \Delta } \sum _{i\in D} \frac{k \mathbf {H}_i\cdot \mathbf {q}+ O(1)}{k \mathbf {X}_i\cdot \mathbf {q}} \xrightarrow [k\rightarrow \infty ]{} \delta _0 {\, \mathop {=}\limits ^{\mathrm {def}}\, }\max _{\mathbf {q}\in \Delta } \sum _{i\in D} \frac{ \mathbf {H}_i\cdot \mathbf {q}}{\mathbf {X}_i\cdot \mathbf {q}}, \end{aligned}$$

where $\Delta$ denotes the space of probability vectors on *J*.

*Estimation of* ${\dim _H}(\Phi )$. Fix a continuous map $\mathbf {z}:\mathbb {R}\rightarrow \Delta$ of period $\rho > 0$, to be determined later. Fix $\gamma > 0$ small, and let $\mathbf {s}:(0,\infty )\rightarrow \Delta$ be defined by the formula

$$\begin{aligned} \mathbf {s}_b = \mathbf {z}(\log (b)/\gamma ). \end{aligned}$$

Next, let $\mathbf {r}_b = \mathbf {M}\cdot \mathbf {s}_b$ for all $b > 0$. Note that $\mathbf {r}$ is exponentially $\lambda$-periodic, where $\lambda = e^{\gamma \rho }$. We will estimate ${\dim _H}(\Phi )$ from below by estimating ${\dim _H}(\nu _{\mathbf {r}})$.

Fix $t\in [0,\rho ]$, and for each $i\in D$ let $B_i > 0$ be given by the formula

8.9 $$\begin{aligned} e^{\gamma t} = \mathbf {X}_i \cdot \mathbf {S}_{B_i}, \end{aligned}$$

where $\mathbf {S}_B {\, \mathop {=}\limits ^{\mathrm {def}}\, }\int _0^B \mathbf {s}_b\;\mathrm {d}b$. Applying (8.7) with $\mathbf {q}= \mathbf {S}_{B_i}$ shows that (2.10) is satisfied with $B = k e^{\gamma t}$. It follows that

$$\begin{aligned} \delta (\mathbf {r},k e^{\gamma t}) = \frac{1}{k e^{\gamma t}} \int h(\{i\in D : b \le B_i\};\mathbf {r}_b)\;\mathrm {d}b. \end{aligned}$$

Now by (8.6),

$$\begin{aligned} h(\{i\in D :b\le B_i\};\mathbf {r}_b)&= \sum _{i:b\le B_i} k\mathbf {H}_i\cdot \mathbf {s}_b + O(1), \end{aligned}$$

and since the left hand side is zero whenever $b > \max _i B_i$, we can add parentheses in the last expression:

$$\begin{aligned} h(\{i\in D : b\le B_i\};\mathbf {r}_b) = \sum _{i:b\le B_i} [k\mathbf {H}_i\cdot \mathbf {s}_b + O(1)]. \end{aligned}$$

So we have

$$\begin{aligned} \delta (\mathbf {r},k e^{\gamma t})&= \frac{1}{k e^{\gamma t}} \int \sum _{i:b\le B_i} [k\mathbf {H}_i\cdot \mathbf {s}_b + O(1)] \;\mathrm {d}b\\&= \frac{1}{ke^{\gamma t}} \sum _{i\in D} \int _0^{B_i} [k\mathbf {H}_i\cdot \mathbf {s}_b + O(1)] \;\mathrm {d}b\\&= \sum _{i\in D} \frac{k\mathbf {H}_i\cdot \mathbf {S}_{B_i} + O(B_i)}{k\mathbf {X}_i\cdot \mathbf {S}_{B_i}}\cdot&\text {(by }(8.9)\text {)} \end{aligned}$$

Since $B_i/\mathbf {X}_i\cdot \mathbf {S}_{B_i}$ is bounded independent of *k*, we have

$$\begin{aligned} \delta (\mathbf {r},k e^{\gamma t}) \xrightarrow [k\rightarrow \infty ]{} \delta (\gamma ;t) {\, \mathop {=}\limits ^{\mathrm {def}}\, }\sum _{i\in D} \frac{\mathbf {H}_i\cdot \mathbf {S}_{B_i}}{\mathbf {X}_i\cdot \mathbf {S}_{B_i}}, \end{aligned}$$

and the convergence is uniform with respect to *t*. So by Theorem 2.11

8.10 $$\begin{aligned} {\dim _H}(\Phi _k) \ge \delta (\mathbf {r}) = \inf _{t\in [0,\rho ]} \delta (\mathbf {r},k e^{\gamma t}) \xrightarrow [k\rightarrow \infty ]{} \delta _\gamma {\, \mathop {=}\limits ^{\mathrm {def}}\, }\inf _{t\in [0,\rho ]} \delta (\gamma ;t). \end{aligned}$$

By (8.8) and (8.10), to complete the proof we must show that $\delta _\gamma > \delta _0$ if $\gamma$ is small enough. It suffices to show that

8.11 $$\begin{aligned} \lim _{\gamma \rightarrow 0} \frac{\delta _\gamma - \delta _0}{\gamma } > 0. \end{aligned}$$

*Taking the limit* $\gamma \rightarrow 0$. In the sequel, we will make the following assumptions about the matrices $\mathbf {H}$ and $\mathbf {X}$:

8.12 $$\begin{aligned}&\text {the maximum in }(8.8) \text { occurs at } \mathbf {q}= \mathbf {u}{\, \mathop {=}\limits ^{\mathrm {def}}\, }(1/3,1/3,1/3), \end{aligned}$$

8.13 $$\begin{aligned}&\mathbf {H}_i\cdot \mathbf {u}= 2^{i - 1} \;\;\forall i, \qquad \qquad \mathbf {X}_i\cdot \mathbf {u}= 2^i \;\;\forall i. \end{aligned}$$

We remark that it follows from these assumptions that $\delta _0 = 3/2$. We also assume that

8.14 $$\begin{aligned} \frac{1}{\rho }\int _0^\rho \mathbf {z}(t) \;\mathrm {d}t = \mathbf {u}\end{aligned}$$

and that $\gamma = \log (2)/(\ell \rho )$ for some $\ell \in \mathbb {N}$. Before proceeding further, let us estimate $\mathbf {S}_{\exp (\gamma t)}^{(\gamma )}$. Here, we have notated the dependence of $\mathbf {S}$ on $\gamma$, since it is relevant to what follows. Let $\mathbf {Z}:\mathbb {R}\rightarrow \mathbb {R}^J$ be the unique antiderivative of $\mathbf {z}- \mathbf {u}$ such that $\int _0^\rho \mathbf {Z}(t)\;\mathrm {d}t = 0$. Note that by (8.14), $\mathbf {Z}$ is periodic of period $\rho$.

### Claim 8.2

We have

8.15 $$\begin{aligned} \mathbf {S}_{\exp (\gamma t)}^{(\gamma )} = e^{\gamma t} [\mathbf {u}+ \gamma \mathbf {Z}(t) + O(\gamma ^2)] \end{aligned}$$

as $$\gamma \rightarrow 0$$.

### Proof

For convenience, we write ${\widehat{\mathbf {z}}}(t) = \mathbf {z}(t) - \mathbf {u}$, ${\widehat{\mathbf {s}}}_b^{(\gamma )} = \mathbf {s}_b^{(\gamma )} - \mathbf {u}$, and ${\widehat{\mathbf {S}}}_B^{(\gamma )} = \mathbf {S}_B^{(\gamma )} - B\mathbf {u}$. Since $\mathbf {s}$ is exponentially $e^{\gamma \rho }$-periodic, we have ${\widehat{\mathbf {S}}}_{\exp (\gamma (t + \rho ))}^{(\gamma )} = e^{\gamma \rho }{\widehat{\mathbf {S}}}_{\exp (\gamma t)}^{(\gamma )}$, so

$$\begin{aligned} {\widehat{\mathbf {S}}}_{\exp (\gamma t)}^{(\gamma )}&= \frac{{\widehat{\mathbf {S}}}_{\exp (\gamma (t + \rho ))}^{(\gamma )} - {\widehat{\mathbf {S}}}_{\exp (\gamma t)}^{(\gamma )}}{e^{\gamma \rho } - 1} = \frac{1}{e^{\gamma \rho } - 1} \int _{e^{\gamma t}}^{e^{\gamma (t + \rho )}} {\widehat{\mathbf {s}}}_b\;\mathrm {d}b\\&= \frac{1}{\gamma \rho + O(\gamma ^2)} \int _0^\rho \gamma e^{\gamma (t + s)} {\widehat{\mathbf {z}}}(t + s)\;\mathrm {d}s\\&= \frac{1}{\rho + O(\gamma )} \int _0^\rho [e^{\gamma (t + s)} - e^{\gamma t}] {\widehat{\mathbf {z}}}(t + s) \;\mathrm {d}s&\text {(by }(8.14)\text {)}\\&= e^{\gamma t} \left[ \frac{\gamma }{\rho } \int _0^\rho s {\widehat{\mathbf {z}}}(t + s)\;\mathrm {d}s + O(\gamma ^2)\right] . \end{aligned}$$

Thus (8.15) holds for the function

8.16 $$\begin{aligned} \mathbf {Z}(t) = \frac{1}{\rho } \int _0^\rho s {\widehat{\mathbf {z}}}(t + s)\;\mathrm {d}s. \end{aligned}$$

Integration by parts shows that $\mathbf {Z}'(t) = {\widehat{\mathbf {z}}}(t)$, and Fubini’s theorem shows that $\int _0^\rho \mathbf {Z}(t)\;\mathrm {d}t = 0$, with both calculations using (8.14). So the function $\mathbf {Z}$ defined by (8.16) is the same as the function $\mathbf {Z}$ defined earlier. $\square$

Let $$t_i = \log (2^i B_i)/\gamma $$. Then

$$\begin{aligned} e^{\gamma (t - t_i)}&= e^{-\gamma t_i}\mathbf {X}_i\cdot \mathbf {S}_{B_i}^{(\gamma )}&\text {(by }(8.9)\text {)}\\&= 2^{-i} e^{-\gamma t_i}\mathbf {X}_i\cdot \mathbf {S}_{\exp (\gamma t_i)}^{(\gamma )}&\text {(since } \log (2) \in \mathbb {N}\gamma \rho \text {)}\\&= 2^{-i} \mathbf {X}_i \cdot \big (\mathbf {u}+ \gamma \mathbf {Z}(t_i) + O(\gamma ^2)\big )&\text {(by }(8.15)\text {)}\\&= 1 + 2^{-i} \gamma \mathbf {X}_i\cdot \mathbf {Z}(t_i) + O(\gamma ^2).&\text {(by }(8.13)\text {)} \end{aligned}$$

In particular $e^{\gamma (t - t_i)} = 1 + O(\gamma )$, which implies that $t - t_i = O(1)$ and thus we can use the Taylor expansion on the left-hand side:

$$\begin{aligned} 1 + \gamma (t - t_i) + O(\gamma ^2) = 1 + \gamma \mathbf {Y}_i\cdot \mathbf {Z}(t_i) + O(\gamma ^2), \end{aligned}$$

where $\mathbf {Y}_i = 2^{-i} \mathbf {X}_i$. Let us write $t_i = t_{i,\gamma }$ to remind ourselves that $t_i$ depends on $\gamma$. We have

$$\begin{aligned} t = t_{i,\gamma } + \mathbf {Y}_i\cdot \mathbf {Z}(t_{i,\gamma }) + O(\gamma ). \end{aligned}$$

So if we let $t_{i,0}\in \mathbb {R}$ be the solution to the equation

$$\begin{aligned} t = t_{i,0} + \mathbf {Y}_i\cdot \mathbf {Z}(t_{i,0}), \end{aligned}$$

then $t_{i,\gamma } = t_{i,0} + O(\gamma )$. This is because the derivative of the right-hand side with respect to $t_{i,0}$ is bounded from below:

$$\begin{aligned} 1 + \mathbf {Y}_i\cdot \mathbf {Z}'(t_{i,0})= & {} \mathbf {Y}_i\cdot \mathbf {u}+ \mathbf {Y}_i\cdot {\widehat{\mathbf {z}}}(t_{i,0})\\= & {} \mathbf {Y}_i\cdot \mathbf {z}(t_{i,0}) \ge \min _{\mathbf {p}\in \Delta } \mathbf {Y}_i\cdot \mathbf {p}= \min _{j\in J} Y_{i,j} > 0. \end{aligned}$$

Next, let $\mathbf {K}_i = 2^{-i}(\mathbf {H}_i - (1/2)\mathbf {X}_i)$. Then

$$\begin{aligned} \frac{\delta (\gamma ;t) - \delta _0}{\gamma }&= \frac{1}{\gamma }\left[ \sum _{i\in D} \frac{\mathbf {H}_i\cdot \mathbf {S}_{B_i}^{(\gamma )}}{\mathbf {X}_i\cdot \mathbf {S}_{B_i}^{(\gamma )}} - \frac{3}{2}\right] \\&= \frac{1}{\gamma }\sum _{i\in D} \frac{\mathbf {K}_i\cdot \mathbf {S}_{B_i}^{(\gamma )}}{\mathbf {Y}_i\cdot \mathbf {S}_{B_i}^{(\gamma )}}\\&= \frac{1}{\gamma }\sum _{i\in D} \frac{\mathbf {K}_i\cdot \mathbf {S}_{2^i B_i}^{(\gamma )}}{\mathbf {Y}_i\cdot \mathbf {S}_{2^i B_i}^{(\gamma )}}&\text {(since }\log (2) \in \mathbb {N}\gamma \rho \text {)}\\&= \frac{1}{\gamma }\sum _{i\in D} \frac{\mathbf {K}_i\cdot \big (\mathbf {u}+ \gamma \mathbf {Z}(t_{i,\gamma }) + O(\gamma ^2)\big )}{\mathbf {Y}_i\cdot \big (\mathbf {u}+ \gamma \mathbf {Z}(t_{i,\gamma }) + O(\gamma ^2)\big )}&\text {(by }(8.15)\text {)}\\&= \frac{1}{\gamma }\sum _{i\in D} \frac{\gamma \mathbf {K}_i\cdot \mathbf {Z}(t_{i,\gamma }) + O(\gamma ^2)}{1 + O(\gamma )}&\text {(by }(8.13)\text {)}\\&= \sum _{i\in D} \mathbf {K}_i\cdot \mathbf {Z}(t_{i,0}) + O(\gamma ) \end{aligned}$$

and thus

$$\begin{aligned} \lim _{\gamma \rightarrow 0}\frac{\delta (\gamma ;t) - \delta _0}{\gamma } = \beta (t) {\, \mathop {=}\limits ^{\mathrm {def}}\, }\sum _{i\in D} \mathbf {K}_i\cdot \mathbf {Z}(t_{i,0}), \end{aligned}$$

and the convergence is uniform with respect to *t*. So to complete the proof, we must show that there exist matrices $\mathbf {H}$ and $\mathbf {X}$ satisfying (8.5), (8.12), and (8.13), such that for some periodic function $\mathbf {z}:\mathbb {R}\rightarrow \Delta$ satisfying (8.14), we have

8.17 $$\begin{aligned} \inf _{t\in [0,\rho ]} \beta (t) > 0. \end{aligned}$$

*Constructing the matrices* $\mathbf {H}$ *and* $\mathbf {X}$; *letting* $\varepsilon \rightarrow 0$. To construct these matrices, let $\mathbf {U}$ be the $3\times 3$ matrix whose entries are all equal to 1, and fix $\varepsilon > 0$ small to be determined. We will let

$$\begin{aligned} \mathbf {H}_i&= 2^i \mathbf {K}_i + (1/2) \mathbf {X}_i,&\mathbf {X}_i&= 2^i \mathbf {Y}_i,&\mathbf {K}&= \varepsilon {\widehat{\mathbf {K}}},&\mathbf {Y}&= \mathbf {U}+ \varepsilon {\widehat{\mathbf {Y}}}, \end{aligned}$$

where ${\widehat{\mathbf {K}}}$ and ${\widehat{\mathbf {Y}}}$ will be chosen later, with the property that

8.18 $$\begin{aligned} {\widehat{\mathbf {K}}}\cdot \mathbf {u}= {\widehat{\mathbf {Y}}}\cdot \mathbf {u}= \mathbf {0}. \end{aligned}$$

Then (8.13) is easily verified, and if $\varepsilon$ is small enough then (8.5) holds. Now for $\mathbf {q}\in \Delta$, we have

8.19 $$\begin{aligned} \sum _{i\in D} \frac{\mathbf {H}_i\cdot \mathbf {q}}{\mathbf {X}_i\cdot \mathbf {q}} - \frac{3}{2}&= \sum _{i\in D} \frac{\mathbf {K}_i\cdot \mathbf {q}}{\mathbf {Y}_i\cdot \mathbf {q}} = \sum _{i\in D} \frac{\varepsilon {\widehat{\mathbf {K}}}_i\cdot \mathbf {q}}{1 + \varepsilon {\widehat{\mathbf {Y}}}_i\cdot \mathbf {q}}\nonumber \\&= \sum _{i\in D} \left[ \varepsilon {\widehat{\mathbf {K}}}_i\cdot \mathbf {q}- \varepsilon ^2 ({\widehat{\mathbf {K}}}_i\cdot \mathbf {q})({\widehat{\mathbf {Y}}}_i\cdot \mathbf {q})\right] + O(\varepsilon ^3 \cdot \Vert \mathbf {q}- \mathbf {u}\Vert ^3). \end{aligned}$$

To demonstrate that (8.12) holds, we need to show that (8.19) is non-positive for all $\mathbf {q}\in \Delta$. To show that this is true whenever $\varepsilon$ is sufficiently small, it suffices to show that

8.20 $$\begin{aligned} \sum _{i\in D} {\widehat{\mathbf {K}}}_i = \mathbf {0} \end{aligned}$$

and

8.21 $$\begin{aligned} \sum _{i\in D} ({\widehat{\mathbf {K}}}_i\cdot \mathbf {q})({\widehat{\mathbf {Y}}}_i\cdot \mathbf {q}) > 0 \;\;\forall \mathbf {q}\in \mathbb {R}^J{\setminus }\mathbb {R}\mathbf {u}. \end{aligned}$$

Finally, to show that (8.17) holds whenever $\varepsilon$ is sufficiently small, we introduce subscripts to indicate the dependence on $\varepsilon$ of all quantities that depend on $\varepsilon$. We have

$$\begin{aligned} t = t_{i,\varepsilon } + \mathbf {Y}_{i,\varepsilon }\cdot \mathbf {Z}(t_{i,\varepsilon }) = t_{i,\varepsilon } + \mathbf {U}_i\cdot \mathbf {Z}(t_{i,\varepsilon }) + \varepsilon {\widehat{\mathbf {Y}}}_i\cdot \mathbf {Z}(t_{i,\varepsilon }). \end{aligned}$$

The middle term is zero, since $\mathbf {U}\cdot {\widehat{\mathbf {z}}}(t) = \mathbf {U}\cdot \mathbf {z}(t) - \mathbf {U}\cdot \mathbf {u}= (1,1,1) - (1,1,1) = \mathbf {0}$ for all $t\in \mathbb {R}$. Thus

$$\begin{aligned} t = t_{i,\varepsilon } + \varepsilon {\widehat{\mathbf {Y}}}_i\cdot \mathbf {Z}(t_{i,\varepsilon }). \end{aligned}$$

So in particular, $t_{i,\varepsilon } = t + O(\varepsilon )$, and thus

$$\begin{aligned} t = t_{i,\varepsilon } + \varepsilon {\widehat{\mathbf {Y}}}_i\cdot \mathbf {Z}(t) + O(\varepsilon ^2). \end{aligned}$$

Thus

$$\begin{aligned} \beta _\varepsilon (t)&= \sum _{i\in D} \mathbf {K}_i\cdot \mathbf {Z}\big (t - \varepsilon {\widehat{\mathbf {Y}}}_i\cdot \mathbf {Z}(t) + O(\varepsilon ^2)\big )\\&= \sum _{i\in D} \varepsilon {\widehat{\mathbf {K}}}_i\cdot \left[ \mathbf {Z}\big (t - \varepsilon {\widehat{\mathbf {Y}}}_i\cdot \mathbf {Z}(t) + O(\varepsilon ^2)\big ) - \mathbf {Z}(t)\right] \quad \text {(by }(8.20)\text {)}\\&= \sum _{i\in D} \big (\varepsilon {\widehat{\mathbf {K}}}_i\cdot \mathbf {Z}'(t)\big )\big (-\varepsilon {\widehat{\mathbf {Y}}}_i\cdot \mathbf {Z}(t)\big ) + O(\varepsilon ^3). \end{aligned}$$

(Note that in this step, we use the fact that $\mathbf {z}$ is continuous (and thus $\mathbf {Z}$ is $C^1$); it is not enough for $\mathbf {z}$ to be piecewise continuous.) So it is enough to show that

8.22 $$\begin{aligned} \sum _{i\in D} \big ({\widehat{\mathbf {K}}}_i\cdot \mathbf {Z}'(t)\big )\big ({\widehat{\mathbf {Y}}}_i\cdot \mathbf {Z}(t)\big ) < 0 \;\;\forall t\in [0,\rho ]. \end{aligned}$$

*Constructing* ${\widehat{\mathbf {K}}}$, ${\widehat{\mathbf {Y}}}$, *and* $\mathbf {z}$. Until now, we have not used the fact that $d = 3$, nor the fact that $\#(D)$ and $\#(J)$ are equal, except as a convenience of notation. But now, we construct explicit matrices ${\widehat{\mathbf {K}}}$ and ${\widehat{\mathbf {Y}}}$ and an explicit continuous periodic function $\mathbf {z}:\mathbb {R}\rightarrow \Delta$ that satisfy (8.14), (8.18), (8.20), (8.21), and (8.22):

$$\begin{aligned} {\widehat{\mathbf {K}}}&= \left[ \begin{array}{ccc} 1 &{}&{} -1\\ -1 &{} 1 &{}\\ &{} -1 &{} 1 \end{array}\right] ,\qquad \qquad {\widehat{\mathbf {Y}}} = \left[ \begin{array}{ccc} 1 &{} -1 &{}\\ &{} 1 &{} -1\\ -1 &{}&{} 1 \end{array}\right] ,\\ \mathbf {z}(t)&= \frac{1}{3}\left( 1 + \cos (t),1 + \cos \left( t + \frac{2\pi }{3}\right) ,1 + \cos \left( t + \frac{4\pi }{3}\right) \right) ,\\ \mathbf {Z}(t)&= \frac{1}{3}\left( \sin (t),\sin \left( t + \frac{2\pi }{3}\right) ,\sin \left( t + \frac{4\pi }{3}\right) \right) . \end{aligned}$$

Now (8.14), (8.18), and (8.20) are immediate. Although it is possible to verify (8.21) and (8.22) by direct computation, we give a geometrical proof. First note that ${\widehat{\mathbf {K}}}$ and ${\widehat{\mathbf {Y}}}$ both commute with the group *G* of orientation-preserving permutation matrices. It follows that the quadratic form $Q_1(\mathbf {q}) = \sum _{i\in D} ({\widehat{\mathbf {K}}}_i\cdot \mathbf {q})({\widehat{\mathbf {Y}}}_i\cdot \mathbf {q})$ is invariant under *G*, and thus the conic section $\{\mathbf {q}\in P: Q_1(\mathbf {q}) = \pm 1\}$ is also invariant under *G*, where *P* is the plane through the origin parallel to $\Delta$, i.e. $P = \{(q_1,q_2,q_3) \in \mathbb {R}^3 : q_1 + q_2 + q_3 = 0\}$. Now if this conic section is a non-circular ellipse, then its major axis must be fixed by *G*, and if it is a hyperbola, then the asymptotes must be either fixed or interchanged. All of these scenarios are impossible because *G* is of order 3 and has no fixed lines in *P*, so the conic section is a circle and thus $Q_1(\mathbf {q}) = c_1 \Vert \mathbf {q}\Vert ^2$ for some constant $c_1$. The sign of $c_1$ can be calculated by taking the trace of $Q_1$, i.e. $3c_1 = \sum _{i\in D} \langle {\widehat{\mathbf {K}}}_i,{\widehat{\mathbf {Y}}}_i\rangle = 3$. Geometrically, this formula is a consequence of the fact that the angle between ${\widehat{\mathbf {K}}}_i$ and ${\widehat{\mathbf {Y}}}_i$ is 60 degrees, and their magnitudes are both $\sqrt{2}$. This demonstrates (8.21).

Next, observe that the path traced by $\mathbf {Z}$ is a circle in *P* centered at the origin, with the opposite orientation from the triangular path $\mathbf {e}_1\rightarrow \mathbf {e}_2\rightarrow \mathbf {e}_3\rightarrow \mathbf {e}_1$.^7^ Thus, for all $t\in \mathbb {R}$ we have $\mathbf {Z}'(t) = \mathbf {v}\times \mathbf {Z}(t)$, where $\times$ denotes the cross product and $\mathbf {v}= -\sqrt{3}\mathbf {u}= -\frac{\sqrt{3}}{3}(1,1,1)$ is a unit vector. So if $\mathbf {N}$ denotes the $3\times 3$ matrix such that $\mathbf {N}\cdot \mathbf {x}= \mathbf {v}\times \mathbf {x}$ for all $\mathbf {x}\in \mathbb {R}^3$, then the left-hand side of (8.22) is equal to

$$\begin{aligned} \sum _{i\in D} \big ({\widehat{\mathbf {K}}}_i \cdot \mathbf {N}\cdot \mathbf {Z}(t)\big ) \big ({\widehat{\mathbf {Y}}}_i\cdot \mathbf {Z}(t)\big ) \end{aligned}$$

and so what is needed is to show that the quadratic form

$$\begin{aligned} Q_2(\mathbf {q}) = \sum _{i\in D} \big ({\widehat{\mathbf {K}}}_i \cdot \mathbf {N}\cdot \mathbf {q}\big ) \big ({\widehat{\mathbf {Y}}}_i\cdot \mathbf {q}\big ) \end{aligned}$$

is negative definite on *P*. Now since $\mathbf {N}$ is a rotation of the plane *P*, it commutes with *G*, so the argument of the preceding paragraph can be used to show that $Q_2(\mathbf {q}) = c_2 \Vert \mathbf {q}\Vert ^2$ for some constant $c_2$ whose sign is the same as the sign of the trace of $Q_2$, i.e. $3c_2 = \sum _{i\in D} \langle {\widehat{\mathbf {K}}}_i\cdot \mathbf {N},{\widehat{\mathbf {Y}}}_i\rangle = -3\sqrt{3}$. Geometrically, this formula is a consequence of the fact that the angle between ${\widehat{\mathbf {K}}}_i\cdot \mathbf {N}$ and ${\widehat{\mathbf {Y}}}_i$ is 150 degrees, and their magnitudes are both $\sqrt{2}$. This demonstrates (8.22). $\square$

### Remark 8.3

It is not hard to see why it is impossible to construct matrices ${\widehat{\mathbf {K}}}$ and ${\widehat{\mathbf {Y}}}$ as well as a periodic function $\mathbf {z}$ satisfying the relevant formulas unless $\#(D),\#(J) \ge 3$. Indeed. if $\#(J) \le 2$, then $\Delta$ is a one-dimensional space, and so by the intermediate value theorem we have $\mathbf {z}(t) = 0$ for some *t*, rendering (8.22) impossible. Similarly, if $\#(D) \le 2$, then by (8.20) we have ${\widehat{\mathbf {K}}}_2 = -{\widehat{\mathbf {K}}}_1$, and again by the intermediate value theorem we have ${\widehat{\mathbf {K}}}_1\cdot \mathbf {z}(t) = 0$ for some *t*. Thus again, (8.22) is impossible in this case.

### Remark 8.4

It should be pointed out that the directions of the inequalities (8.21) and (8.22) are irrelevant to the question of whether there exist ${\widehat{\mathbf {K}}}$, ${\widehat{\mathbf {Y}}}$, and $\mathbf {z}$ satisfying them. Indeed, if ${\widehat{\mathbf {K}}}$ (or ${\widehat{\mathbf {Y}}}$) is replaced by its negative, then the signs of both inequalities simultaneously flip, while if $\mathbf {z}$ is replaced by the function $t\mapsto \mathbf {z}(-t)$, then the sign of (8.22) flips but the sign of (8.21) stays the same. So given a triple $({\widehat{\mathbf {K}}},{\widehat{\mathbf {Y}}},\mathbf {z})$ that satisfies (8.21) and (8.22) with respect to any given direction of signs, it is possible to modify this triple in a minor way to get a triple that satisfies (8.21) and (8.22) with respect to the correct direction of signs.

## Open questions

Although Theorem 2.8 provides an answer to Question 1.2 in dimensions 3 and higher, it is natural to ask what happens in dimension 2:

### Questions 9.1

If $X \subset \mathbb {R}^2$ is a compact set and $T:X\rightarrow X$ is an expanding map satisfying the specification property, then is the Hausdorff dimension of *X* equal to the supremum of the Hausdorff dimensions of the ergodic *T*-invariant measures? And if so, is the supremum attained, and what are the properties of the measure attaining the supremum? What if the specification property is not assumed?

Although we have proven that the dimension gap ${\dim _H}(\Phi ) - {\dim _D}(\Phi )$ is strictly positive, we cannot get a very good lower bound on its size. This leads to some natural questions:

### Questions 9.2

Given $d \ge 3$, what is

$$\begin{aligned} \text {MDG}(d) {\, \mathop {=}\limits ^{\mathrm {def}}\, }\sup _{\Lambda _\Phi \subset [0,1]^d} \big ({\dim _H}(\Phi ) - {\dim _D}(\Phi )\big ), \end{aligned}$$

where the supremum is taken over all Barański sponges $\Lambda _\Phi$? (Here MDG is short for “maximal dimension gap”.) Is the answer any different if the supremum is restricted to sponges that satisfy the coordinate ordering condition? And what about the related quantity

$$\begin{aligned} \text {MDG}'(d) {\, \mathop {=}\limits ^{\mathrm {def}}\, }\sup _{\Lambda _\Phi \subset [0,1]^d} \frac{{\dim _H}(\Phi ) - {\dim _D}(\Phi )}{{\dim _D}(\Phi )}? \end{aligned}$$

In our proofs it seems that this quantity is more natural to consider than $\text {MDG}(d)$; for example, we can show that $\text {MDG}'(d) \le d - 2$ for all $d \ge 2$ (Theorem 7.2 above). To avoid the effects of low dimension, we ask: what is the asymptotic behavior of $\text {MDG}'(d)$ as $d \rightarrow \infty$? For example, is it bounded or unbounded?

Although Theorem 2.9 shows that the map $\Phi \mapsto {\dim _H}(\Phi )$ is continuous on the space of Barański sponges, in many contexts the Hausdorff dimension is not only continuous but real-analytic (see e.g. [1, 41, 47–49]). So we ask:

### Questions 9.3

Is the function $\Phi \mapsto {\dim _H}(\Phi )$ real-analytic, or at least piecewise real-analytic, on the space of Barański sponges? What about the subclass of strongly Barański sponges?

Finally, we speculate that the key ideas behind our definition of a pseudo-Bernoulli measure might apply more generally. We therefore ask the following questions:

### Questions 9.4

Is there any useful class of measures that exhibits scale-dependent behavior similar to pseudo-Bernoulli measures in a more general context? For example, can the ideas of this paper be used to construct repellers with a dimension gap other than sponges?

## Footnotes

1 Read literally, the setup of [3] implies that the maps $\phi _{i,a}$ ($i\in D$, $a\in A_i$) are orientation-preserving, but there is no significant difference in dealing with the case where reflections are allowed.

2 This terminology is not meant to imply that the Lyapunov exponents are distinct or have been arranged in increasing order, although it is often convenient to assume the latter (cf. Proposition 2.16 below).

3 Specifically, it is not clear whether every expanding repeller can be embedded into an expanding global endomorphism of a compact manifold.

4 The variance of $-\log \mathbf {r}_n([\omega _n]_I)$ is at most $\#(E) \max _{x\in [0,1]} x\log ^2(x)$.

5 This is called Corollary 1.8 in the appendix of the preprint version of [11].

6 Here *O*(1) denotes a quantity whose magnitude is bounded by a constant, in particular independent of *k*.

7 Although we have checked that the signs and orientations in this paragraph are correct (and we thank the referee for pointing out a couple of errors in a previous version), it is not necessary to check this to verify the validity of the argument; cf. Remark 8.4.
